# Patient-reported outcomes in oropharyngeal cancer: comparing two cohorts with different treatment protocols

**DOI:** 10.1007/s00520-026-10763-2

**Published:** 2026-05-13

**Authors:** Helena Ullgren, Per Fransson, Ylva Tiblom Ehrsson, Göran Laurell

**Affiliations:** 1 Department of Oncology-Pathology, Karolinska Institute, Stockholm, Sweden; 2 Theme Cancer Head and Neck, Lung and Skin Cancer, Karolinska Comprehensive Cancer Center, Stockholm, Sweden; 3 Department of Nursing, Umeå University, Umeå, Sweden; 4 Department of Surgical Sciences; Otorhinolaryngology and Head and Neck Surgery, Uppsala University, Uppsala, Sweden

**Keywords:** Oropharyngeal cancer, Patient-reported outcome measures, Radiotherapy techniques, Total radiotherapy dose and adjuvant treatment

## Abstract

**Purpose:**

Long-term patient-reported outcomes (PROMs) may provide insight into the impact of advances in the treatment of oropharyngeal squamous cell carcinoma (OPSCC). The main aim of the study was to assess and compare PROMs in OPSCC patients treated during two different periods (1998–2006 vs. 2015–2021), reflecting advances in treatment strategies and developments in care.

**Methods:**

The present study is based on data from two Swedish multicentre studies: the randomised controlled trial ARTSCAN1 (1998–2006) and the observational study NIPHNC (2015–2021). Patients received curative-intent radiotherapy as part of the ARTSCAN or NIPHNC studies. All patients completed PROMs at multiple time points up to 24 months post-treatment. PROMs were assessed using the European Organisation for Research and Treatment of Cancer (EORTC) Quality of Life Questionnaire EORTC QLQ-H&N35 and the Hospital Anxiety and Depression Scale HADS.

**Results:**

A total of 363 patients were included, with 161 and 202 patients from ARTSCAN1 and NIPHNC, respectively. At baseline and at all post-radiotherapy time points, the NIPHNC cohort reported fewer symptoms and lower anxiety/depression. At 2 years, both groups showed improvement, though persistent issues like dry mouth and sticky saliva remained common.

**Conclusions:**

Comparison of two treatment protocols demonstrates improvements in short- and long-term PROMs over time. Long-term salivary symptoms remain prevalent in patients with OPSCC.

**Supplementary Information:**

The online version contains supplementary material available at 10.1007/s00520-026-10763-2.

## Introduction

Oropharyngeal squamous cell carcinoma (OPSCC) is, during recent decades, probably the most well-studied type of head and neck squamous cell carcinoma (HNSCC). OPSCC can be classified as human papillomavirus (HPV)-negative (HPV-) and HPV-positive (HPV +) [1, 2].

Treatment modalities include radiotherapy (RT), chemoradiation (CRT) and surgery, with external beam RT and CRT being safe and effective modalities. Transoral Robotic Surgery (TORS) has been implemented in many countries, especially in high-volume tertiary care centres [3]. Cervical nodal involvement is common in OPSCC, requiring a thorough neck evaluation before treatment, as nodal positivity negatively impacts outcomes [4, 5]. Various neck treatment approaches have been explored previously. In patients with node-negative OPSCC undergoing RT or CRT as a first-line treatment, the neck is usually electively irradiated [6, 7]. The surgical approach can be either elective or therapeutic neck dissection (ND) [8, 9].

Both single-modality RT and combined-modality treatment carry the risk of side effects. The assessment of long-term advancements in treatment has been underrepresented in studies. During the last few decades, several developments promoting improvements in patients with OPSCC have been identified in the surgical, radiotherapeutic, and medical oncology fields [10–13]. It has been postulated that the use of newer RT techniques to combat OPSCC, such as intensity-modulated RT (IMRT) and proton therapy, produces better local control and fewer severe side effects than those of 3D conformal RT (3DCRT) [14–17]. We hypothesised that patients with OPSCC treated during two different time periods would report differences in long-term side effects. In this study, patient-reported outcome measures (PROMs) were compared between two cohorts treated during 1998–2006 and 2015–2021.

## Materials and methods

### Design

We compared two cohorts of patients with OPSCC from two Swedish prospective multicentre studies on HNSCC: the Accelerated RT of squamous cell carcinomas (ARTSCAN1) study [18–20] and the Nutrition and Inflammation in patients with HNSCC Cancer (NIPHNC) study (ID NCT03343236 in ClinicalTrials.gov, 2017–11–10) [21, 22]. We followed the STROBE (Strengthening the Reporting of Observational Studies in Epidemiology) [23] guidelines, and a completed STROBE checklist is provided as supplementary material.

The ARTSCAN1 study is a randomised controlled trial that enrolled 750 patients with HNSCC from 12 Swedish RT centres between November 1998 and June 2006. The primary aim of this study was to compare treatment outcomes of RT administered as accelerated or conventional fractionation schedules. 

The NIPHNC study is an observational multicentre study in which 522 participants were included between November 2015 and December 2022 from three tertiary-referral HNSCC cancer centres in Sweden. This study aimed to investigate the relationship between inflammation and nutritional status. Inclusion and exclusion criteria for both studies are detailed in Johansson et al.[20] and Astradsson et al.[22].

Only patients diagnosed with OPSCC from the ARTSCAN1 and NIPHNC studies were included in the present study. This study was approved by the Swedish Ethical Review Authority (No. 2023—02561—02) and conducted in accordance with the Declaration of Helsinki.

### Treatment

The inclusion criteria for ARTSCAN1 patients were a histology-proven OPSCC diagnosis and treatment with 3DCRT at 2 Gray (Gy)/day, up to 68 Gy over 7 weeks. Treatment could be administered as a single-modality or as preoperative RT, with or without systemic anti-cancer treatment (SACT). ND could be performed as a part of the RT treatment protocol [19].

The criteria for NIPHNC patients were a histology-proven OPSCC diagnosis and treatment with IMRT or volumetric intensity-modulated arc therapy (VMAT) with 2 Gy/fraction, up to 68–70 Gy over 7 weeks, with or without SACT. In selected cases, RT was followed by ND approximately 3 months after the end of RT (e-RT), based on a positive fluorodeoxyglucose positron emission tomography/computed tomography (FDG PET/CT) scan.

### Data collection

In the ARTSCAN1 and NIPHNC studies, PROMs were collected before RT (baseline) and at the end of RT (e-RT). In both studies, informed consent was obtained from all patients before participation. Follow-up assessments were conducted at 6 months, 1 year, and 2 years after the completion of RT. The ARTSCAN1 patients completed the baseline questionnaire at the hospital, and the following questionnaires were sent by mail with a prepaid envelope. One reminder was sent if needed.

In the NIPHNC study, a research nurse contacted the patients at all follow-ups via email or phone. Questionnaires were administered in both digital and paper formats, depending on patient preference. One reminder was sent if needed.

PROM data were reported from baseline until the 2-year follow-up or until recurrence or death.

### Patient characteristics and clinical variables

Patient characteristics and clinical variables included age, sex, smoking status, blood sample of haemoglobin (Hb), tumour stage (UICC 7), RT techniques (3DCRT, IMRT/VMAT), total RT dose (< 66 Gy or ≥ 66 Gy), with/without SACT and ND post-RT.

### Instruments

To assess PROM, both the European Organisation for Research and Treatment of Cancer (EORTC) Quality of Life Questionnaire–H&N35 (EORTC QLQ-H&N35) [24, 25] and the Hospital Anxiety and Depression Scale (HADS) were used [26]. The EORTC QLQ-H&N35 comprises 35 items divided into seven multiple-item symptom scales, six single-item symptom scales, and five no/yes items. Responses range from one to four, representing "not at all", "a little", "quite a lot", and "a lot". For the no/yes items, the scores indicate the percentage of “yes” responses.

Symptoms of anxiety and depression were measured using the HADS, which included 14 items. The items were divided into two subscales: Anxiety (HADS-A) and Depression (HADS-D), each with seven items. Each item was scored on a 4-point Likert scale (0–3), with both subscale scores ranging from 0 to 21. Scores of 0–7 indicate normal levels, 8–10 suggest borderline cases, and 11–21 indicate clinically significant anxiety or depression [26].

### Data analysis

The item scores of the EORTC QLQ-H&N35 were transformed into a 0–100 scale [27]. Scale scores were calculated, with higher scores on the symptom scales representing more symptoms. Osoba et al., 1998 [28] set thresholds for interpreting clinically relevant differences in scores as follows: small (≥5–10), moderate (>10–20), and large clinically relevant differences (>20). Descriptive statistics and proportions were calculated for binary variables, while continuous variables were summarised with minimum, maximum, median, and mean values.

The Chi-square test was used to analyse differences in categorical variables, and the Mann–Whitney U-test was used for continuous variables.

Linear regression (both univariate and multivariable) analyses were performed to examine variables associated with differences in symptom scores between the two groups. First, univariate linear regression analyses were performed for each symptom on the QLQ-H&N35 and the HADS scales, using all patients as the sample. In the second step, independent variables (smoker, RT technique, sex, age, stage high/low, neck dissection and SACT) associated with the dependent variable (p ≤ 0.1) were included in separate multivariable linear regression analyses for each dependent factor.

To further describe the differences in symptoms between the groups at the 2-year follow-up, responses were dichotomised by combining 1 and 2 (not at all and a little) and 3 and 4 (quite a lot and a lot) into two groups. All reported p-values were based on a two-sided hypothesis, with p < 0.05 indicating statistical significance. IBM SPSS v. 29.0.1.0 (171) was used for the analysis.

## Results

### Patients, clinical characteristics, treatment, and PROMs at baseline

In total, 363 patients met the eligibility criteria for this study: 161 in the ARTSCAN1 group and 202 in the NIPHNC group. Clinical characteristics, and treatment details are summarised in Table 1. Sex distribution, disease stage, and haemoglobin levels showed no differences between the groups. HPV status was unavailable for a substantial subset of cases and was therefore excluded from the table*.*

**Table 1:** Clinical and treatment characteristics of patients with oropharyngeal squamous cell carcinoma

Characteristic	Total cohort (*n* = 363)	ARTSCAN1 (*n* = 161)	NIPHNC (*n* = 202)	*P*-value*
Sex, *n* (%)				0.616
Male, *n* (%)	280 (77.2)	122 (75.8)	158 (78.2)	
Female, *n* (%)	83 (22.9)	39 (24.2)	44 (21.8)	
Age, years, mean (range)	60 (35–86)	59 (37–86)	62 (35–85)	< 0.001
Stage (%)				0.283
Stage I-II	34 (9.4)	12 (7.5)	22 (10.9)	
Stage III-IV	329 (90.6)	149 (41.0)	180 (49.6)	
Smoking, *n* (%) **	83 (24.9)	48 (29.8)	35 (17.3)	<0.001
Missing, *n* (%)		30 (18.6)	7 (3.5)	
Haemoglobin, mean (range)	140 (96–171)	140 (100–168)	140 (96–171)	0.920
Missing, *n* (%)	140 (96–171)	37 (23)	7 (3.5)	
Radiotherapy (RT) dose < 66 Gy, *n* (%)	73 (21.0)	70 (45.5)	3 (1.6)	<0.001
Dose ≥ 66 Gy	281 (77.4)	85 (52.7)	196 (97)	
Missing	9 (2.5)	6 (3.7)	3 (1.5)	
Neck dissection (ND), *n* (%)	100 (27.5)	81 (50.3)	19 (9.4)	<0.001
Missing, *n* (%)		1 (0.6)	2 (1.0)	
Time from RT end to ND	65 (27–141)	55 (27–113)	106 (49–141)	<0.001
In days, mean (range)				
Systemic anti-cancer treatment (SACT) total, *n* (%)	104 (28.7)	16 (9.9)	88 (43.6)	< 0.001

*Chi-square test for categorical variables and t-test for continuous variables

**Both former and current smokers

However, differences were observed in age, smoking status, RT dose < 66 Gy and ≥ 66 Gy, ND rates, time from RT end to ND, and SACT administration (Table 1). ND was more frequent in the ARTSCAN1 group (50.3% vs. 9.4%). During the 2-year follow-up, 32 patients (19.9%) in the ARTSCAN1 group died, compared to 13 (6.4%) in the NIPHNC group. Furthermore, 41 patients developed locoregional and/or distant recurrence and were excluded from further assessment. Table 2 shows the number of eligible patients who responded to the questionnaire at each time point. One and seven patients in the ARTSCAN1 and NIPHNC groups, respectively, had no baseline assessment; however, they were included in the analysis because they completed the questionnaires at follow-up.

**Table 2:** Number of patients with oropharyngeal squamous cell carcinoma completing the questionnaires at baseline and at each follow-up

Time point	ARTSCAN1 (*n* = 161)	NIPHNC (*n* = 202)
Baseline	160/161 (99%)	195/202 (97%)
End of radiotherapy	152/158 (96%)	179/200 (90%)
6 months	138/150 (92%)	168/193 (87%)
1 year	116/124 (94%)	163/184 (89%)
2 years	109/110 (99%)	**126/143 (88%)

*The total in each group included eligible patients at each time point, excluding those who experienced recurrence or died

**20 patients in the NIPHNC group have not reached the 2-year follow-up

**Fig. 1:**
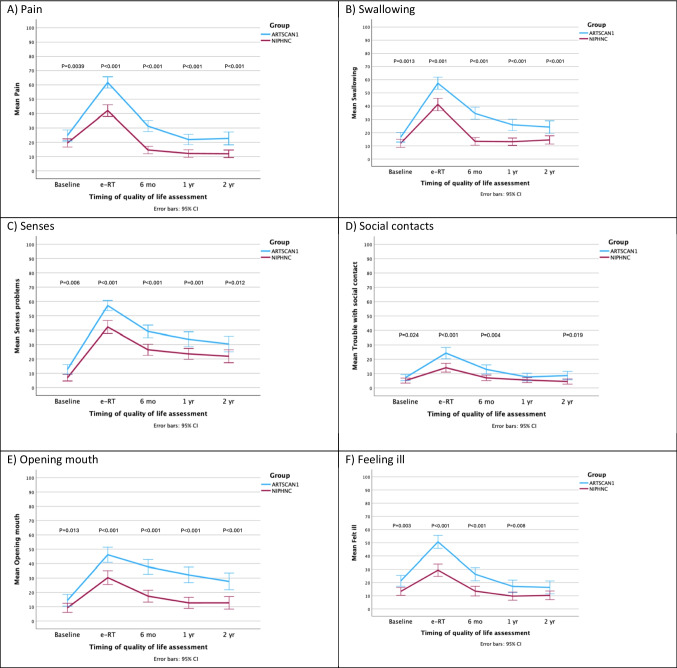
**A**–**F** HRQoL scores for six symptoms, at baseline and at each follow-up reported by the ARTSCAN1 and NIPHNC groups. Mean values are based on responses from patients who completed the EORTC QLQ-H&N35. Higher scores indicate more severe symptoms or impairments. Only significant P-values are reported

**Fig. 2:**
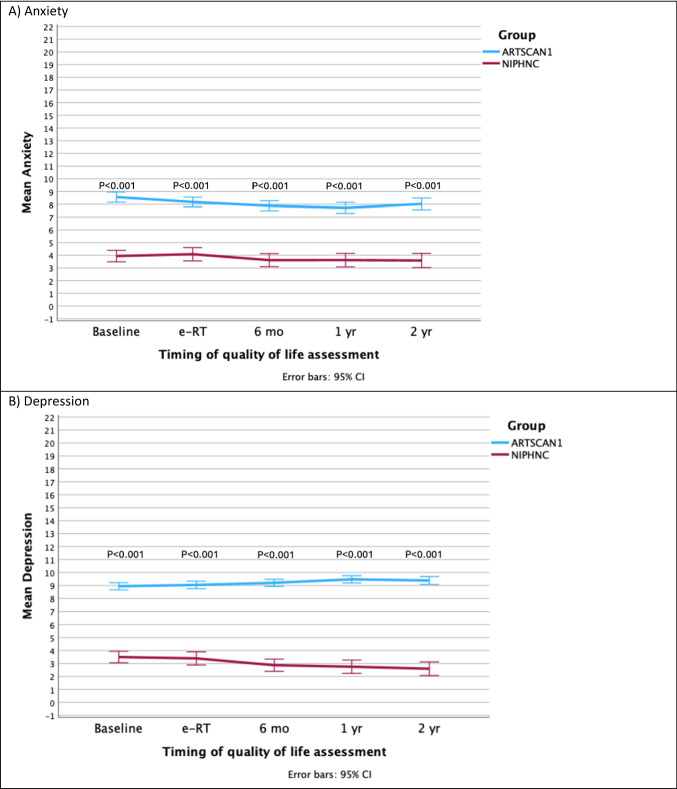
**A**-**B** Scores from the HADS questionnaire were collected from the ARTSCAN1 and NIPHNC groups at baseline and at each follow-up. Higher scores indicate more symptoms of anxiety (**A**) and depression (**B**)

Regarding PROMs at baseline, differences were observed in six out of 18 symptoms (Fig. 1A-F) Small but clinically relevant differences were observed for senses and mouth opening, while a moderate difference was observed for feeling ill (Table 3), all indicating higher symptom burden in the ARTSCAN1 group. At baseline, the ARTSCAN1 group reported higher mean scores of anxiety (8.6 vs. 4.0) and depression (8.9 vs. 3.5) than those of the NIPHNC group (p < 0.001) (Fig. 2A-B).

**Table 3:** Differences in the mean EORTC H&N35 scores between the ARTSCAN1 and NIPHNC groups at baseline, the end of radiotherapy (e-RT), and the 2-year follow-up

Scale	Group	Mean scores at baseline	Difference in scores at baseline	*P* value	Mean scores at the eRT	Difference in scores at the eRT	*P* value	Mean scores at the 2-year follow-up	Difference in scores at the 2-year follow-up	*P* value*
Pain	ARTSCAN1	24.8	**5.3**	**0.039**	61.7	**19.7**	** < 0.001**	22.7	10.7*	** < 0.001**
	NIPHNC	19.5			42.0			12.0		
Swallowing	ARTSCAN1	16.6	4.6	0.013	57.3	**16.0**	** < 0.001**	24.3	**9.7**	** < 0.001**
	NIPHNC	12.0			41.3			14.6		
Senses problems	ARTSCAN1	12.8	**5.9**	**0.006**	57.3	**15.0**	** < 0.001**	30.5	**8.5**	**0.012**
	NIPHNC	6.9			42.3			22.0		
Speech problems	ARTSCAN1	13.2	0	0.958	46.6	**17.4**	** < 0.001**	18.2	**7.1**	**0.055**
	NIPHNC	13.2			29.2			11.1		
Trouble with social eating	ARTSCAN1	17.6	4.4	0.111	57.3	**16.4**	** < 0.001**	26.9	**13.9**	** < 0.001**
	NIPHNC	13.2			40.9			13.0		
Trouble with social contact	ARTSCAN1	7.6	2.5	**0.024**	24.3	**10.2**	** < 0.001**	8.6	4.1	< 0.019
	NIPHNC	5.1			14.1			4.5		
Less sexuality	ARTSCAN1	30.1	2.9	0.365	61.8	**10.0**	**0.024**	34.1	**9.4**	**0.029**
	NIPHNC	27.2			51.8			24.7		
Teeth	ARTSCAN1	18.4	6.3	0.078	28.2	**13.8**	** < 0.001**	34.6	**15.7**	** < 0.001**
	NIPHNC	12.1			14.4			18.9		
Open mouth	ARTSCAN1	14.5	**5.3**	**0.013**	46.1	**15.8**	** < 0.001**	27.7	**15.0**	** < 0.001**
	NIPHNC	9.2			30.3			12.7		
Dry mouth	ARTSCAN1	23.4	2.7	0.178	71.5	**14.6**	** < 0.001**	75.7	**26.0**	** < 0.001**
	NIPHNC	20.7			56.9			49.7		
Sticky saliva	ARTSCAN1	22.3	4.7	0.138	83.2	**16.3**	** < 0.001**	55.7	**22.6**	** < 0.001**
	NIPHNC	17.4			66.9			33.1		
Coughing	ARTSCAN1	20.1	3.4	0.129	43.6	4.1	0.211	24.7	3.8	0.173
	NIPHNC	16.7			39.5			20.9		
Felt ill	ARTSCAN1	26.1	**12.8**	**0.003**	50.6	**21.2**	** < 0.001**	16.4	6.1	0.090
	NIPHNC	13.3			29.4			10.3		
Painkillers	ARTSCAN1	53.2	0.4	0.929	82.2	7.8	0.092	27.5	−2.5	0.680
	NIPHNC	53.6			74.4			30.0		
Nutritional supplements	ARTSCAN1	19.0	1.8	0.663	78.3	**14.3**	**0.005**	19.3	1.0	0.857
	NIPHNC	17.2			64.0			18.3		
Feeding tube	ARTSCAN1	23.5	7.5	0.132	41.7	**19.7**	** < 0.001**	6.4	**5.6**	**0.022**
	NIPHNC	16.0			22.0			0.8		
Weight loss	ARTSCAN1	44.2	−1.1	0.642	75.2	10.2	0.052	12.8	4.5	0.267
	NIPHNC	45.3			65.0			8.3		
Weight gain	ARTSCAN1	35.7	5.5	0.178	9.7	−3.8	0.306	21.3	−7.0	0.221
	NIPHNC	30.2			13.5			28.3		

* Statistically and clinically significant differences are marked in bold

### Comparisons of PROMS at the end of radiotherapy

Differences were observed between the two groups in 14 of the 18 HRQoL symptoms at the e-RT. All symptoms showed moderate clinically relevant differences, except for feeling ill, which demonstrated a large clinically relevant difference. In all cases, the NIPHNC group had more favourable outcomes, experiencing fewer or less severe symptoms, than those of the ARTSCAN1 group (Table 3; Fig. 1A-F. Supplementary Material, Fig. 1G-X).

The ARTSCAN1 group reported higher mean scores for anxiety (8.2 vs. 3.4) and depression (9.0 vs. 4.1) than those of the NIPHNC group (*p* < 0.001) (Fig. 2A and B). 

**Fig. 3:**
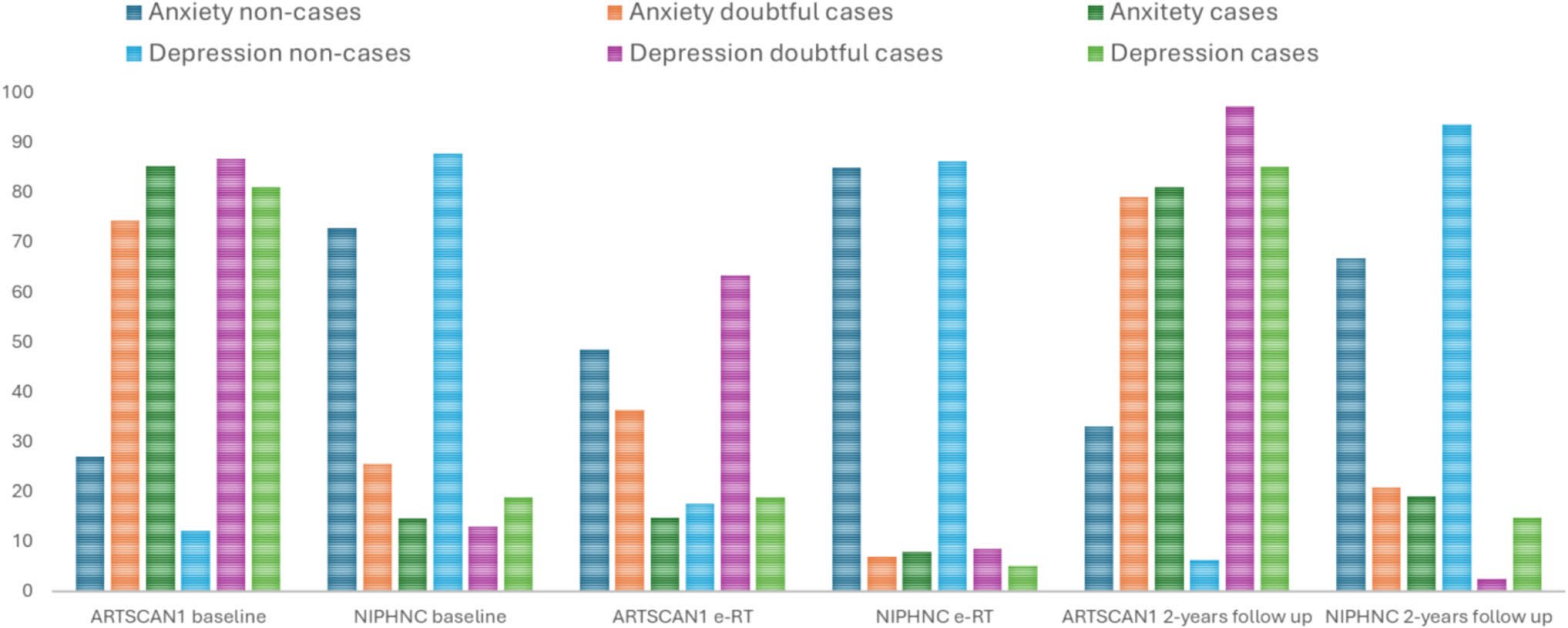
Proportions of anxiety and depression according to HADS (non-cases, doubtful cases, and confirmed cases) in the ARTSCAN1 and NIPHNC groups at baseline, end of radiotherapy (e-RT), and 2-year follow-up

The proportion of doubtful and confirmed cases of anxiety and depression was higher in the ARTSCAN1 group than in the NIPHNC group (Fig. 3).

A multivariable linear regression analysis at the e-RT demonstrated that 8 of the 18 HRQoL symptoms were associated with at least one of the independent variables: age (high), stage (high), SACT, sex (male), and smoker. Further, SACT were associated with higher anxiety scores, while age (high) and SACT were associated with higher depression scores (Table 1, Supplementary material).

### Comparisons of PROMS at 2-year follow-up

Six symptoms exhibited moderate clinically relevant differences, while dry mouth and sticky saliva showed large clinically relevant differences (Table 3; Fig. 1A, 1C, 1K, 1P, 1N, 1Q; Supplementary Material).

**Fig. 4:**
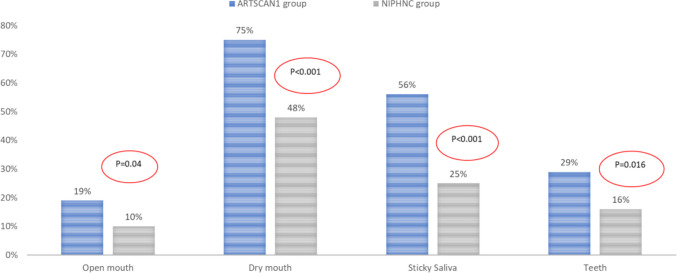
Proportions of “Quite a lot” and/or “A lot” for single-item symptoms (open mouth, dry mouth, sticky saliva and teeth) from the EORTC QLQ-HN35 questionnaire in the ARTSCAN1 and NIPHNC groups at the 2-year follow-up

The symptoms, which showed a moderate-to-large clinically relevant difference in mean scores between the two groups at the 2-year follow-up, were analysed further by comparing the proportions of patients reporting “quite a lot and-/or “a lot” at two years (Fig. 4). The symptom scores were dichotomised to illustrate clinically relevant differences between the two groups. A higher proportion of patients reporting “quite a bit” and “very much” was observed in the ARTSCAN1 group than in the NIPHNC group.

A similar pattern was observed in the proportion of non-cases, doubtful cases, and cases of anxiety and depression, with differences between the two groups at both baseline and 2-year follow-up (Fig. 3).

At the 2-year follow-up, multivariable linear regression analysis revealed that stage (high), smoker, SACT and ND were associated with 4 of the 18 EORTC QLQ-HN35 scales (speech problems, trouble with social eating, dry mouth and weight loss). SACT were associated with both anxiety and depression, and age (high) *was* associated with depression (Supplementary material, Table 1).

## Discussion

The primary treatments for OPSCC include RT, SACT, and surgery, with RT being the cornerstone treatment for decades. This retrospective study describes the changes in PROM over time, in two cohorts of OPSCC patients undergoing different treatment protocols and care strategies during two distinct periods.

A modern approach to assessing treatment outcomes in patients with cancer places PROM as a central measure alongside traditional metrics, such as survival and recurrence rates. Patients with OPSCC frequently experience significant ongoing challenges, as many of their needs for symptom relief, psychosocial support, and comprehensive rehabilitation remain insufficiently addressed [29, 30]. Given the high overall survival rate of patients with HPV-positive OPSCC [8, 31], assessing long-term follow-up of PROM is essential. Therefore, we adopted a pragmatic approach to compare two distinct cohorts from studies with markedly different designs conducted in 1998–2006 and 2015–2021. Although the two studies had different objectives, all patients underwent robust two-year surveillance with repeated PROM measurements. Notwithstanding that those patients had completed the same questionnaires, the EORTC QLQ-H&N35 and HADS, comparisons between the studies may still be affected by inherent limitations. Despite these limitations, we believe that comparing the two cohorts from different time periods provides valuable insight into the evolution of treatment strategies for OPSCC. Moreover, a randomised controlled trial directly comparing the treatment modalities used in the present study (3DCRT versus IMRT/VMAT) would not be ethically justifiable.

In addition to differences in RT techniques, this study identified baseline and treatment-related differences between the two groups. In the ARTSCAN1 group, ND was performed as a standard surgical procedure in 50% of patients, with a mean of 55 days after the e-RT. In contrast, the NIPHNC group adopted a more selective approach in 9% of patients, performing ND at a mean of 106 days after the e-RT, based on positive follow-up PET/CT findings. Furthermore, during the earlier treatment period (1998–2006), more extensive neck dissection techniques were generally used, both in elective and therapeutic settings, whereas in the later period (2015–2021), neck dissection was mainly performed for therapeutic indications. The multivariable analysis suggests that ND contributes to differences in PROM outcomes (dry mouth) between the two cohorts at 2-year follow-up.

Additionally, the NIPHNC group showed a shift towards a higher total RT dose and more frequent use of systemic anti-cancer therapy than did the ARTSCAN1 group. In addition to the changes in treatment described above, several other changes in the care of patients with OPSCC in Sweden may have affected the results. These include the implementation of a standardised pathway to minimise the time from diagnosis to treatment, access to support and information by a specialised nurse with in-depth knowledge responsible for coordination and support (contact nurse) [32, 33], and the introduction of an individualised care plan (ICP) [34, 35]. Although the comparison revealed several important differences between the two treatment protocols and care, multivariable analysis did not identify any consistent pattern that could explain the improvement in most PROMs observed in the latter cohort.

At baseline, minor differences were observed; however, a moderate clinically-relevant difference was observed for feeling ill, favouring the NIPHNC group. Additionally, the ARTSCAN1 group showed worse anxiety and depression scores than those of the NIPHNC group, suggesting higher baseline psychological distress, which could affect both treatment adherence and recovery. We speculate that a possible explanation for the baseline differences in the levels of anxiety and depression could be the recent advancements in the care of patients with OPSCC, for example, the standardised pathways (to minimise the time between suspected diagnosis and treatment), mentioned above. Another important consideration is that this study is based on historical data, and the two cohorts were treated at different time periods. Consequently, they were exposed to different background prevalences of HPV‑associated disease in the population. The ARTSCAN1 cohort was likely treated at a time when HPV‑related disease was less common, while the more recent NIPHNC cohort was treated when HPV‑associated OPSCC was more prevalent.

These differences may have affected how patients were informed regarding their prognosis. We therefore suggest that the differences seen in baseline anxiety and depression may partly reflect changes in the information patients received about their prognosis—patients in the NIPHNC cohort were more likely to be told they had a favourable outlook.

At the e-RT, PROM data were collected at a standardised time point following the completion of RT for all patients, at a stage when no ND had been performed. This timing ensured that any differences in the reported symptoms were not due to recovery time, allowing for a more direct comparison of the effects of each primary treatment approach. In the entire study population, most of the symptoms worsened during treatment. However, the cohort treated later (the NIPHNC group) had fewer symptoms than those observed in the ARTSCAN1 group.

Although there were differences in the RT technique, dosing, and adjuvant treatment, both groups of patients demonstrated similar and parallel recovery in PROM over the two years following treatment. Gradual improvement was observed in both groups, with the largest changes occurring during the first six months after the e-RT. Thus, tracking PROM over two years indicated that both groups of recurrence-free survivors exhibited resilience, effectively adapting to and recovering from treatment. At the two-year follow-up, large clinically relevant differences [28] between the two groups were observed only for sticky saliva and dry mouth. In contrast, moderate clinically relevant differences were noted for four additional symptoms. Dry mouth and sticky saliva are two persistent symptoms that have also been reported previously, including one study in which patients expressed a need for better support [36]. However, it is important to note that despite improvements, half of the patients in the NIPHNC group continued to report significant problems with dry mouth at the two-year follow-up.

One key conclusion is that the differences in PROM between the two treatment periods are likely multifactorial, reflecting the rising prevalence of HPV-associated disease, changes in treatment and care, and potential differences in aggressiveness between HPV-negative and HPV-positive disease. Several comparative studies have shown that IMRT/VMAT provides moderately superior sparing of organs-at-risk (OARs) compared to 3DCRT [37]. This improved side-effect profile has been the primary driver for adopting IMRT over 3DCRT in clinical practice. In HNSCC, IMRT/VMAT has been found to better protect OARs, including the spinal cord, oral cavity, carotid arteries, and pharyngeal constrictors, than does 3DCRT [38, 39]. Moreover, studies have frequently shown that IMRT reduces the risk of xerostomia by sparing the salivary glands, particularly the parotid glands, minimising the dose and reducing long-term side effects [40]. This aligns with the positive patient-reported outcomes in the NIPHNC group, which included fewer problems with dry mouth and sticky saliva. Our findings are consistent with the trial data from a previous study on patients with nasopharyngeal cancer [41].

Few studies have investigated PROM outcomes across two distinct treatment periods, and most previous evaluations of 3DCRT and IMRT/VMAT have overlooked the influence of adjuvant therapies.

Although the results suggest that IMRT/VMAT may offer better symptom relief, there is no consensus that these two techniques significantly improve the recurrence rate or overall survival in OPSCC [17, 42].

Regarding symptoms of anxiety and depression, differences were observed at baseline, making comparisons during and after treatment challenging. Throughout the entire two-year follow-up period, the ARTSCAN1 group exhibited higher anxiety and depression scores. When comparing the proportions of non-cases, doubtful cases, and confirmed cases, the NIPHNC group showed more favourable outcomes.

The strengths of this study include a relatively large cohort, comprehensive data on adjuvant treatment from the two study periods, and the original prospective design applied to both patient groups. Additionally, response rates for the questionnaires were high, strengthening the reliability of our PROM assessment. However, this study has some limitations. Baseline differences may have influenced the outcomes, and additionally, key variables, such as HPV, p16 expression, treatment target volume, and dosimetric parameters, were not available. Moreover, variations in clinical care and surgical techniques for ND cannot be excluded as potential contributors to the results. The absence of clinical and treatment-related variables, together with baseline differences between the cohorts, limits the generalisability of the results. Although the original studies had markedly different designs and included some heterogeneity between the two study populations, the present study highlights that, over time, treatment paradigms have improved and contributed to enhanced patient-reported outcomes. Future studies should aim to address these limitations by incorporating a more comprehensive dataset, including HPV status, p16 expression, treatment target volume, and dosimetric parameters, to better account for potential confounders.

## Conclusions

Treatment protocols across the two distinct periods had different impacts on PROMs in patients with OPSCC. The latter cohort (NIPHNC) demonstrated more favourable levels of PROMs throughout and experienced less severe late side effects. For most symptoms, the greatest difference was observed at the end of primary treatment. The improvements in short- and long-term PROMs in OPSCC treatment may be attributed to several factors, including HPV prevalence, evolving treatment strategies and advancements in patient care. Nevertheless, patients in both cohorts reported considerable problems with dry mouth and sticky saliva at the two-year follow-up. Persistent issues like dry mouth and sticky saliva require proactive management strategies. Enhanced supportive care, including salivary substitutes, oral hydration protocols, and emerging interventions, should be considered to improve patient quality of life in patients with OPSCC.

## Supplementary Information

Below is the link to the electronic supplementary material.

Supplementary file1 (DOCX 4305 kb)

Supplementary file2 (DOCX 56 kb)

## Data Availability

The data are not publicly available due to privacy and ethical restrictions.

## References

[1] Ndon S, Singh A, Ha PK, Aswani J, Chan JY, Xu MJ (2023) Human papillomavirus-associated oropharyngeal cancer: global epidemiology and public policy implications. Cancers (Basel). 10.3390/cancers15164080 37627108 10.3390/cancers15164080 PMC10452639

[2] Cui Z, Kang H, Li H, Lee ED, Lee YS, Peterson CN, Long SR, Grandis JR, Johnson DE (2024) CYLD alterations are associated with metastasis and poor prognosis in human papilloma virus-positive head and neck cancer. Head Neck. 10.1002/hed.27944 39347568 10.1002/hed.27944 PMC11845079

[3] Faraji F, Kumar A, Voora R, Soliman SI, Cherry D, Courtney PT, Finegersh A, Guo T, Cohen E, Califano JA III, Mell L, Rose B, Orosco RK (2024) Transoral surgery in HPV-positive oropharyngeal carcinoma: oncologic outcomes in the Veterans Affairs system. Laryngoscope 134:207–214 37255050 10.1002/lary.30784 PMC10687307

[4] Alexiev BA, Obeidin F, Johnson DN, Finkelman BS, Prince R, Somani SN, Cheng E, Samant S (2020) Oropharyngeal carcinoma: a single institution study of 338 primaries with special reference to high-risk human papillomavirus-mediated carcinoma with aggressive behavior. Pathol Res Pract 216:153243 33113454 10.1016/j.prp.2020.153243

[5] Abdel-Halim CN, O’Byrne TJ, Graves JP, Akpala CO, Moore EJ, Price DL, Tasche KT, Ma DJ, Neben-Wittich MA, Lester SC, Gamez M, Price KA, Bayne HEF, Rwigema JCM, Patel SH, McGee LA, Janus JR, Nagel TH, Hinni ML, Savvides PS, Van Abel KM, Routman DM (2023) Patterns and distribution of regional nodal involvement and recurrence in a surgically treated oropharyngeal squamous cell carcinoma cohort at a tertiary center. Oral Oncol 146:106569 37734203 10.1016/j.oraloncology.2023.106569

[6] Pursley J, Damato AL, Czerminska MA, Margalit DN, Sher DJ, Tishler RB (2017) A comparative study of standard intensity-modulated radiotherapy and RapidArc planning techniques for ipsilateral and bilateral head and neck irradiation. Med Dosim 42:31–36 27919621 10.1016/j.meddos.2016.10.004

[7] Embring A, Onjukka E, Mercke C, Lax I, Berglund A, Friesland S (2023) Dose escalation in oropharyngeal cancer: a comparison of simultaneous integrated boost and brachytherapy boost. Radiat Oncol 18:65 37029424 10.1186/s13014-023-02256-x PMC10082532

[8] Mehanna H, Taberna M, von Buchwald C, Tous S, Brooks J, Mena M, Morey F, Grønhøj C, Rasmussen JH, Garset-Zamani M, Bruni L, Batis N, Brakenhoff RH, Leemans CR, Baatenburg de Jong RJ, Klussmann JP, Wuerdemann N, Wagner S, Dalianis T, Marklund L, Mirghani H, Schache A, James JA, Huang SH, O’Sullivan B, Nankivell P, Broglie MA, Hoffmann M, Quabius ES, Alemany L, Mehanna H, Taberna M, von Buchwald C, Tous S, Huang SH, O’Sullivan B, Garset-Zamani M, Brooks J, Batis N, Fulton-Lieuw T, Nankivell P, Schache A, James JA, Brakenhoff RH, Leemans CR, Heideman DAM, Bloemena E, Nauta I, de Jong RB, Dalianis T, Marklund L, Mirghani H, Wagner S, Wittekindt C, Klussmann JP, Wuerdemann N, Quaas A, Sharma SJ, Maltseva M, Zimmermann P, Hoffmann M, Quabius ES, Däppen MB, Ärztin L, Bruni L, Mena M, Morey F, Alemany L (2023) Prognostic implications of p16 and HPV discordance in oropharyngeal cancer (HNCIG-EPIC-OPC): a multicentre, multinational, individual patient data analysis. Lancet Oncol 24:239–251 36796393 10.1016/S1470-2045(23)00013-X

[9] Marur S, D’Souza G, Westra WH, Forastiere AA (2010) HPV-associated head and neck cancer: a virus-related cancer epidemic. Lancet Oncol 11:781–789 20451455 10.1016/S1470-2045(10)70017-6 PMC5242182

[10] Verellen D, Vanhavere F (1999) Risk assessment of radiation-induced malignancies based on whole-body equivalent dose estimates for IMRT treatment in the head and neck region. Radiother Oncol 53:199–203 10660198 10.1016/s0167-8140(99)00079-1

[11] de Bree R, van der Putten L, Brouwer J, Castelijns JA, Hoekstra OS, Leemans CR (2009) Detection of locoregional recurrent head and neck cancer after (chemo)radiotherapy using modern imaging. Oral Oncol 45:386–393 19095487 10.1016/j.oraloncology.2008.10.015

[12] Ferris MJ, Danish H, Switchenko JM, Deng C, George BA, Goldsmith KC, Wasilewski KJ, Cash WT, Khan MK, Eaton BR, Esiashvili N (2017) Favorable local control from consolidative radiation therapy in high-risk neuroblastoma despite gross residual disease, positive margins, or nodal involvement. Int J Radiat Oncol Biol Phys 97:806–812 28244417 10.1016/j.ijrobp.2016.11.043 PMC5502807

[13] Calvert M, Kyte D, Mercieca-Bebber R, Slade A, Chan AW, King MT, the S-PROG, Hunn A, Bottomley A, Regnault A, Chan AW, Ells C, O’Connor D, Revicki D, Patrick D, Altman D, Basch E, Velikova G, Price G, Draper H, Blazeby J, Scott J, Coast J, Norquist J, Brown J, Haywood K, Johnson LL, Campbell L, Frank L, von Hildebrand M, Brundage M, Palmer M, Kluetz P, Stephens R, Golub RM, Mitchell S, Groves T (2018) Guidelines for inclusion of patient-reported outcomes in clinical trial protocols: the SPIRIT-PRO extension. JAMA 319:483–494 29411037 10.1001/jama.2017.21903

[14] Wang X, Eisbruch A (2016) IMRT for head and neck cancer: reducing xerostomia and dysphagia. J Radiat Res 57(Suppl 1):i69–i75 27538846 10.1093/jrr/rrw047 PMC4990117

[15] Chen JL, Huang YS, Kuo SH, Hong RL, Ko JY, Lou PJ, Wang CW (2017) Intensity-modulated radiation therapy achieves better local control compared to three-dimensional conformal radiation therapy for T4-stage nasopharyngeal carcinoma. Oncotarget 8:14068–14077 27764778 10.18632/oncotarget.12736 PMC5355163

[16] Blanchard P, Gunn GB, Lin A, Foote RL, Lee NY, Frank SJ (2018) Proton therapy for head and neck cancers. Semin Radiat Oncol 28:53–63 29173756 10.1016/j.semradonc.2017.08.004

[17] Alterio D, Gugliandolo SG, Augugliaro M, Marvaso G, Gandini S, Bellerba F, Russell-Edu SW, De Simone I, Cinquini M, Starzynska A, Zaffaroni M, Bacigalupo A, Fanetti G, Durante S, Dicuonzo S, Orecchia R, Jereczek-Fossa BA (2021) IMRT versus 2D/3D conformal RT in oropharyngeal cancer: a review of the literature and meta-analysis. Oral Dis 27:1644–1653 32810381 10.1111/odi.13599

[18] Nyqvist J, Fransson P, Laurell G, Hammerlid E, Kjellen E, Franzen L, Soderstrom K, Wickart-Johansson G, Friesland S, Sjodin H, Brun E, Ask A, Nilsson P, Ekberg L, Bjork-Eriksson T, Nyman J, Loden B, Lewin F, Reizenstein J, Lundin E, Zackrisson B (2016) Differences in health related quality of life in the randomised ARTSCAN study; accelerated vs. conventional radiotherapy for head and neck cancer. A five year follow up. Radiother Oncol 118:335–341 26777124 10.1016/j.radonc.2015.12.024

[19] Zackrisson B, Nilsson P, Kjellen E, Johansson KA, Modig H, Brun E, Nyman J, Friesland S, Reizenstein J, Sjodin H, Ekberg L, Loden B, Mercke C, Fernberg JO, Franzen L, Ask A, Persson E, Wickart-Johansson K, Lewin F, Wittgren L, Bjor O, Bjork-Eriksson T (2011) Two-year results from a Swedish study on conventional versus accelerated radiotherapy in head and neck squamous cell carcinoma--the ARTSCAN study. Radiother Oncol 100:41–48 21295880 10.1016/j.radonc.2010.12.010

[20] Johansson KA, Nilsson P, Zackrisson B, Ohlson B, Kjellen E, Mercke C, Alvarez-Fonseca M, Billstrom A, Bjork-Eriksson T, Bjor O, Ekberg L, Friesland S, Karlsson M, Lagerlund M, Lundkvist L, Lofroth PO, Lofvander-Thapper K, Nilsson A, Nyman J, Persson E, Reizenstein J, Rosenbrand HO, Wiklund F, Wittgren L (2008) The quality assurance process for the ARTSCAN head and neck study - a practical interactive approach for QA in 3DCRT and IMRT. Radiother Oncol 87:290–299 18206256 10.1016/j.radonc.2007.12.005

[21] Einarsson S, Karlsson HE, Bjor O, Haylock AK, Tiblom Ehrsson Y (2020) Mapping impact factors leading to the GLIM diagnosis of malnutrition in patients with head and neck cancer. Clin Nutr ESPEN 40:149–155 33183529 10.1016/j.clnesp.2020.09.174

[22] Astradsson T, Sellberg F, Ehrsson YT, Sandstrom K, Laurell G (2022) Serum proteomics in patients with head and neck cancer: peripheral blood immune response to treatment. Int J Mol Sci. 10.3390/ijms23116304 35682983 10.3390/ijms23116304 PMC9180944

[23] von Elm E, Altman DG, Egger M, Pocock SJ, Gøtzsche PC, Vandenbroucke JP (2007) The Strengthening the Reporting of Observational Studies in Epidemiology (STROBE) statement: guidelines for reporting observational studies. Ann Intern Med 147:573–577 17938396 10.7326/0003-4819-147-8-200710160-00010

[24] Aaronson NK, Ahmedzai S, Bergman B, Bullinger M, Cull A, Duez NJ, Filiberti A, Flechtner H, Fleishman SB, de Haes JC et al (1993) The European Organization for Research and Treatment of Cancer QLQ-C30: a quality-of-life instrument for use in international clinical trials in oncology. J Natl Cancer Inst 85:365–376 8433390 10.1093/jnci/85.5.365

[25] Bjordal K, Hammerlid E, Ahlner-Elmqvist M, de Graeff A, Boysen M, Evensen JF, Biörklund A, de Leeuw JR, Fayers PM, Jannert M, Westin T, Kaasa S (1999) Quality of life in head and neck cancer patients: validation of the European Organization for Research and Treatment of Cancer Quality of Life Questionnaire-H&N35. J Clin Oncol 17:1008–1019 10071296 10.1200/JCO.1999.17.3.1008

[26] Zigmond AS, Snaith RP (1983) The hospital anxiety and depression scale. Acta Psychiatr Scand 67:361–370 6880820 10.1111/j.1600-0447.1983.tb09716.x

[27] Fayers P, Bottomley A, Group EQoL, Quality of Life U (2002) Quality of life research within the EORTC-the EORTC QLQ-C30. European Organisation for Research and Treatment of Cancer European journal of cancer 38(Suppl 4):S125-133 11858978 10.1016/s0959-8049(01)00448-8

[28] Osoba D, Rodrigues G, Myles J, Zee B, Pater J (1998) Interpreting the significance of changes in health-related quality-of-life scores. J Clin Oncol 16:139–144 9440735 10.1200/JCO.1998.16.1.139

[29] McDowell L, Casswell G, Bressel M, Drosdowsky A, Rischin D, Coleman A, Shrestha S, D’Costa I, Fua T, Tiong A, Liu C, Gough K (2021) Symptom burden, quality of life, functioning and emotional distress in survivors of human papillomavirus associated oropharyngeal cancer: an Australian cohort. Oral Oncol 122:105560 34653749 10.1016/j.oraloncology.2021.105560

[30] Busca I, Giuliani ME, Weiss J, Jones J, Quartey NK, Huang SH, Toulany A, Papadakos J, Ringash JG (2022) Long term results of a longitudinal study of unmet survivorship needs in patients with head and neck cancer. Int J Radiat Oncol Biol Phys 112:e56–e57

[31] Lauritzen BB, Gronlund MW, Jakobsen KK, Justesen MM, Garset-Zamani M, Carlander AF, Rasmussen JH, Bendtsen SK, Kiss K, Andersen G, Rosenorn MR, Friborg J, Bentzen JKD, Gronhoj C (2020) von Buchwald C (2024) Epidemiological trends and survival of oropharyngeal cancer in a high HPV-prevalent area: a Danish population-based study from 2000 to. Int J Cancer 155:2169–2179 10.1002/ijc.35099 39016028

[32] Westman B, Kirkpatrick L, Ebrahim F, Henriksson R, Sharp L (2018) Patient-reported experiences on supportive care strategies following the introduction of the first Swedish national cancer strategy and in accordance with the new patient act. Acta Oncol 57:382–392 29276836 10.1080/0284186X.2017.1418089

[33] Westman B, Ullgren H, Olofsson A, Sharp L (2019) Patient-reported perceptions of care after the introduction of a new advanced cancer nursing role in Sweden. Eur J Oncol Nurs Off J Eur Oncol Nurs Soc 41:41–48 10.1016/j.ejon.2019.05.009 31358256

[34] Granstrom B, Isaksson J, Westoo N, Holmlund T, Tano K, Laurell G, Tiblom Ehrsson Y (2023) Perceptions of life and experiences of health care support among individuals one year after head and neck cancer treatment - an interview study. Eur J Oncol Nurs 66:102383 37506610 10.1016/j.ejon.2023.102383

[35] Talani C, Hogmo A, Laurell G, Makitie A, Farnebo L (2024) Six-month mortality has decreased for patients with curative treatment intent for head and neck cancer in Sweden. PLoS One 19:e0296534 38625920 10.1371/journal.pone.0296534 PMC11020944

[36] Kanatas A, Lowe D, Rogers SN (2022) The Patient Concerns Inventory in head and neck oncology: a structured review of its development, validation and clinical implications. Eur Arch Otorhinolaryngol 279:5097–5111 35842858 10.1007/s00405-022-07499-0 PMC9519723

[37] Citrin DE (2017) Recent developments in radiotherapy. N Engl J Med 377:1065–1075 28902591 10.1056/NEJMra1608986

[38] Eisbruch A (2016) Can xerostomia be further reduced by sparing parotid stem cells? Ann Transl Med 4:S16 27867984 10.21037/atm.2016.10.25 PMC5104650

[39] Upadhyay R, Liao K, Grosshans DR, McGovern SL, McAleer FM, Zaky W, Chintagumpala MM, Mahajan A, Nana Yeboa D, Paulino AC (2022) Quantifying the risk and dosimetric variables of symptomatic brainstem injury after proton beam radiation in pediatric brain tumors. Neuro Oncol 24:1571–1581 35157767 10.1093/neuonc/noac044 PMC9435496

[40] Bisof V, Rakusic Z, Bibic J, Grego T, Soce M (2018) Comparison of intensity modulated radiotherapy with simultaneous integrated boost (IMRT-SIB) and a 3-dimensional conformal parotid gland-sparing radiotherapy (ConPas 3D-CRT) in treatment of nasopharyngeal carcinoma: a mono-institutional experience. Radiol Med 123:217–226 29094268 10.1007/s11547-017-0824-9

[41] L J-C, H J-M, J Y-M, L D-W, C C-M, L C-S, H W-Y, S Y-F, L K-T, F C-Y, L C-H, C H-L (2014) Comparisons of quality of life for patients with nasopharyngeal carcinoma after treatment with different RT technologies. Acta Otorhinolaryngol Ital 34:241–246 25210217 PMC4157538

[42] Kawamoto T, Nihei K, Nakajima Y, Kito S, Sasai K, Karasawa K (2018) Comparison of xerostomia incidence after three-dimensional conformal radiation therapy and contralateral superficial lobe parotid-sparing intensity-modulated radiotherapy for oropharyngeal and hypopharyngeal cancer. Auris Nasus Larynx 45:1073–1079 29397249 10.1016/j.anl.2018.01.010

