# Supplementary material for: Patient-reported outcomes in oropharyngeal cancer: comparing two cohorts with different treatment protocols
Source: Support Care Cancer. 2026 May 13;34(6):536. doi: 10.1007/s00520-026-10763-2 (PMC13171772; doi:10.1007/s00520-026-10763-2)
Supplement: Supplementary file 1 — Supplementary file1 (DOCX 4305 kb) [file 520_2026_10763_MOESM1_ESM.docx]

**Appendix A Supplementary material**

| 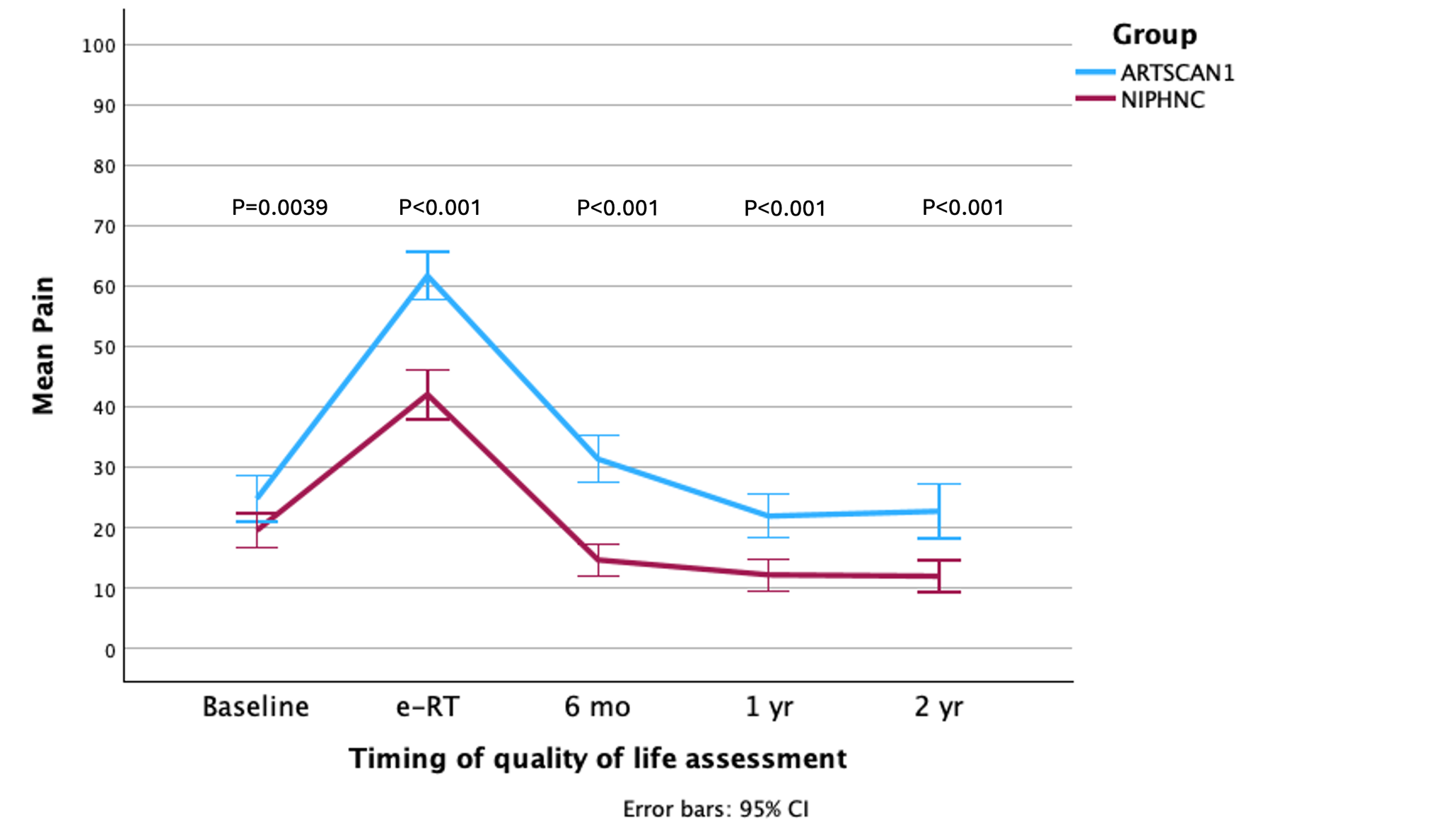  Figure 1G. Symptom scales of the EORTC QLQ- H&N35 (0-100 point scale) from baseline over 2 years reported by the ARTSCAN1 and NIPHNC groups. Mean values based on patients answering the questionnaire. Higher scores indicate more severe symptoms or impairments. Only significant P-values are reported. |
| --- |
| 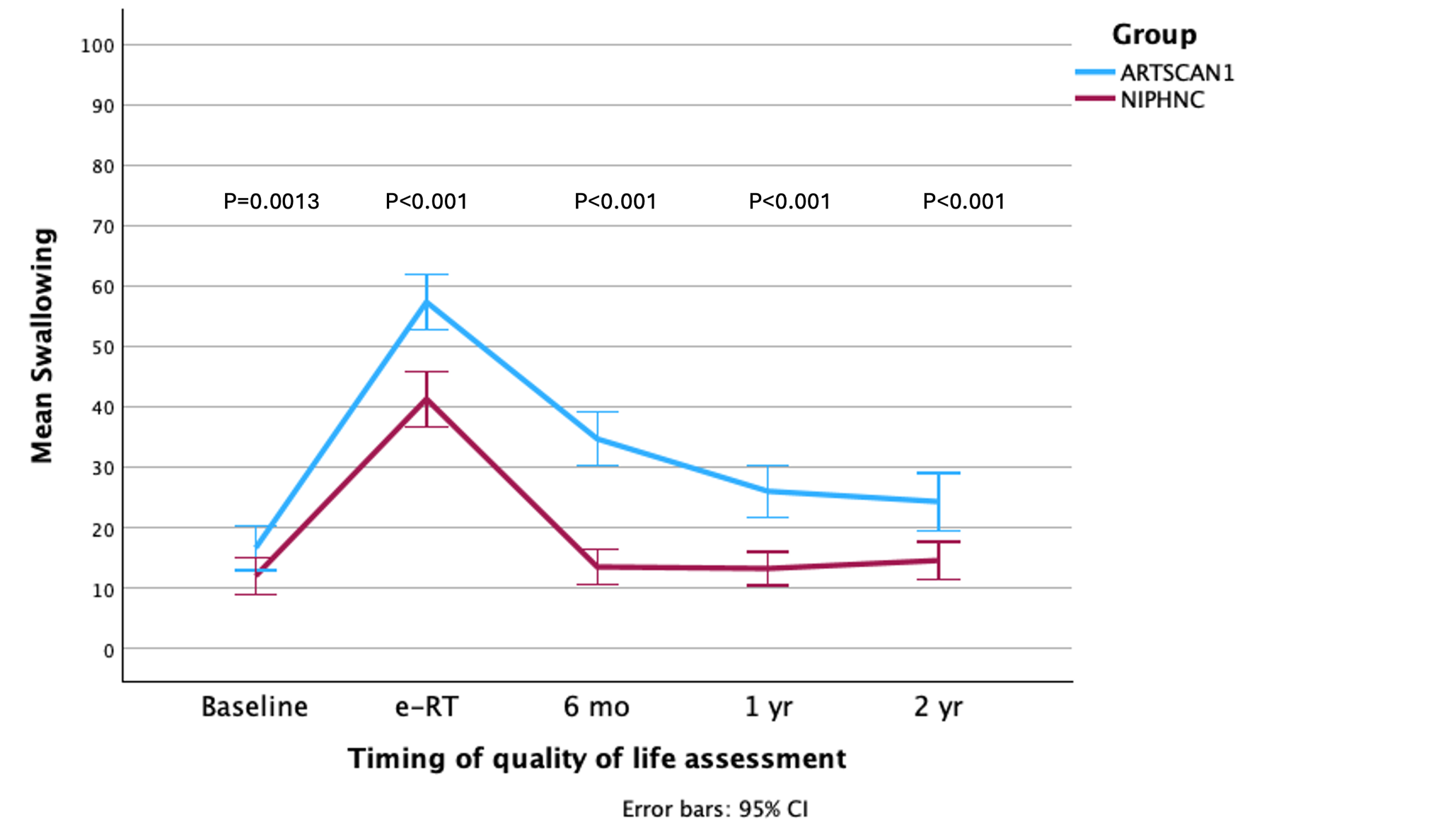  Figure 1H. Symptom scales of the EORTC QLQ- H&N35 (0-100 point scale) from baseline over 2 years reported by the ARTSCAN1 and NIPHNC groups. Mean values based on patients answering the questionnaire. Higher scores indicate more severe symptoms or impairments. Only significant P-values are reported. |
| 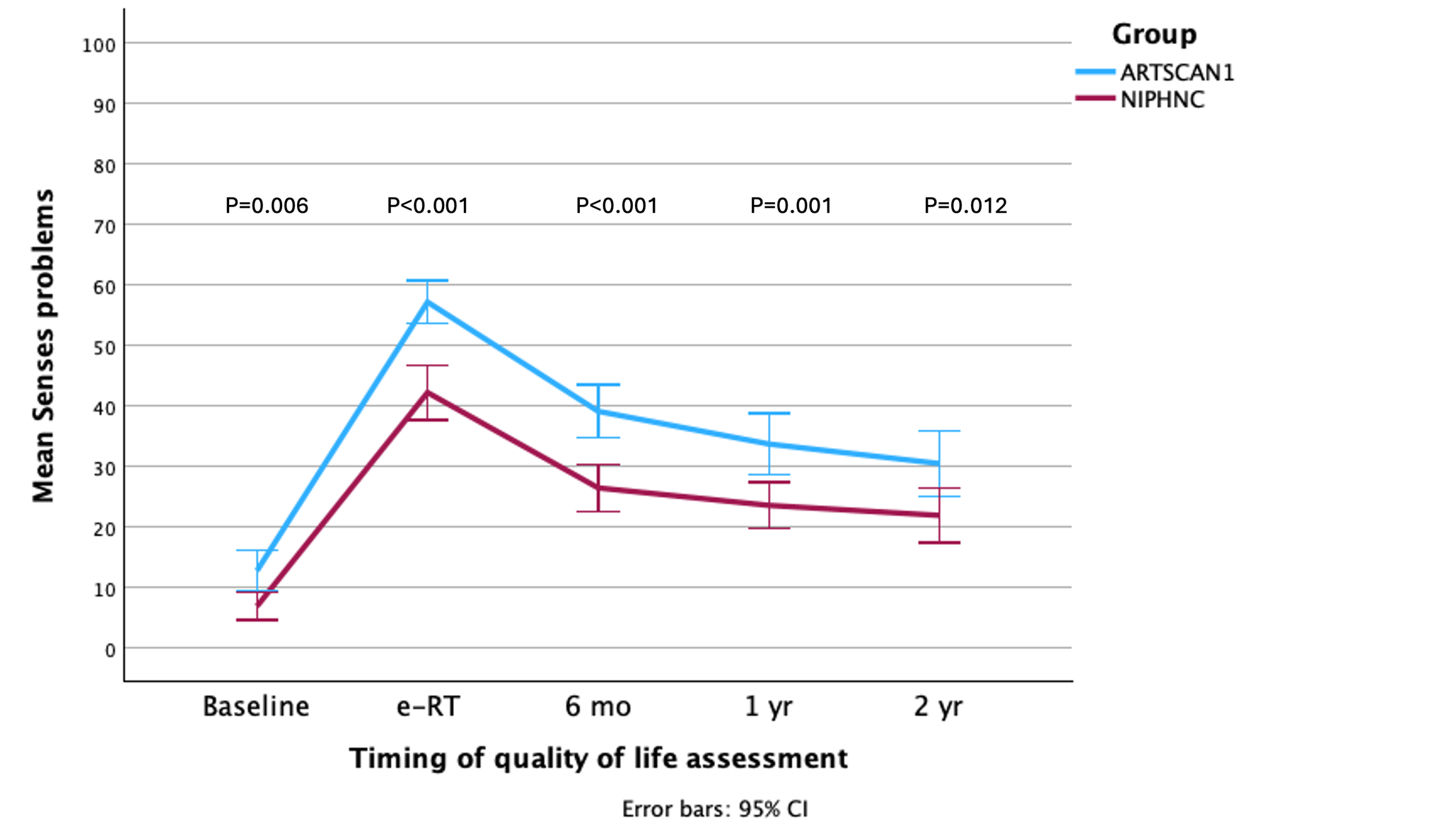  Figure 1I. Symptom scales of the EORTC QLQ- H&N35 (0-100 point scale) from baseline over 2 years reported by the ARTSCAN1 and NIPHNC groups. Mean values based on patients answering the questionnaire. Higher scores indicate more severe symptoms or impairments. Only significant P-values are reported. |
| 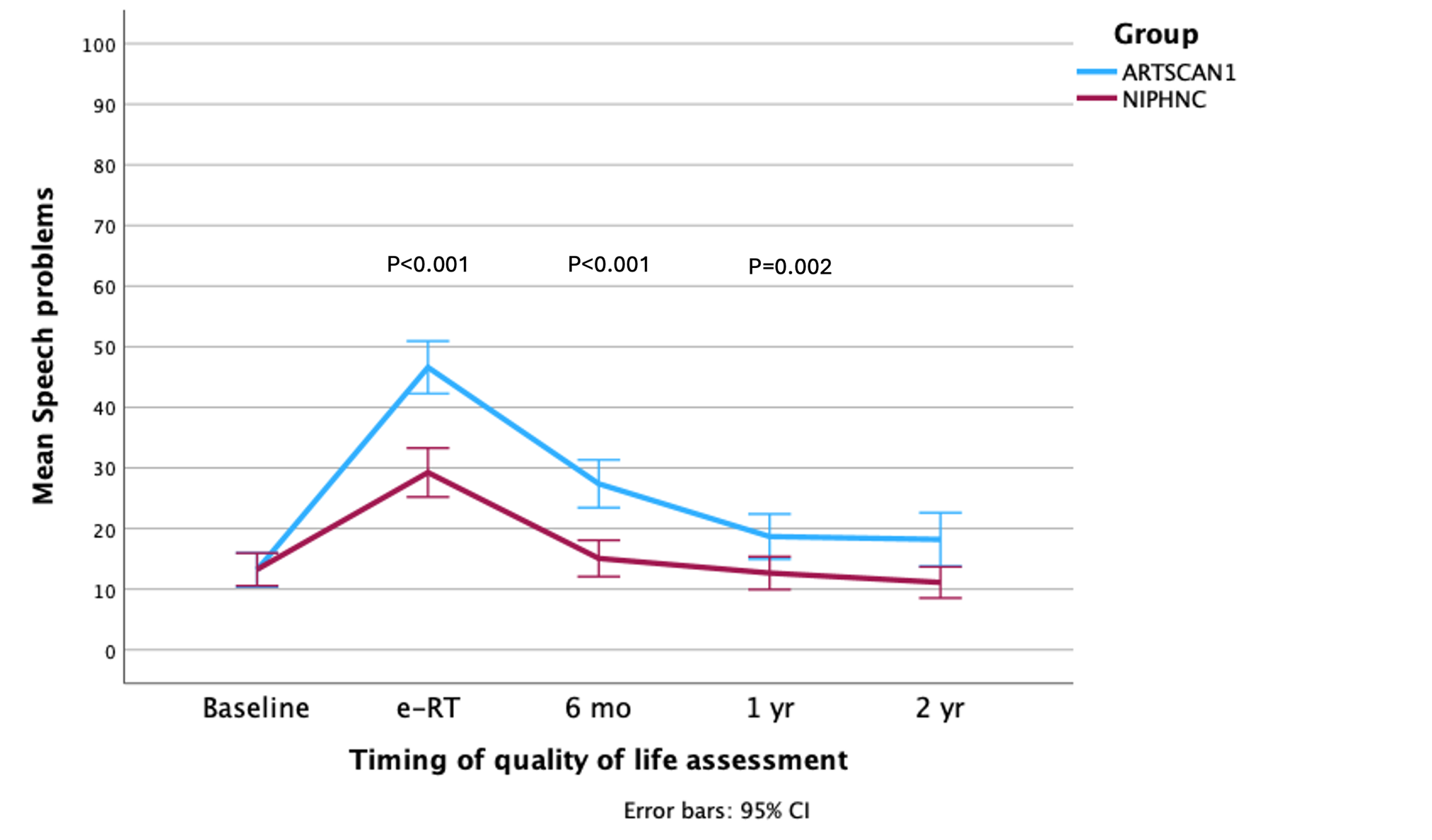  Figure 1J. Symptom scales of the EORTC QLQ- H&N35 (0-100 point scale) from baseline over 2 years reported by the ARTSCAN1 and NIPHNC groups. Mean values based on patients answering the questionnaire. Higher scores indicate more severe symptoms or impairments. Only significant P-values are reported. |
| 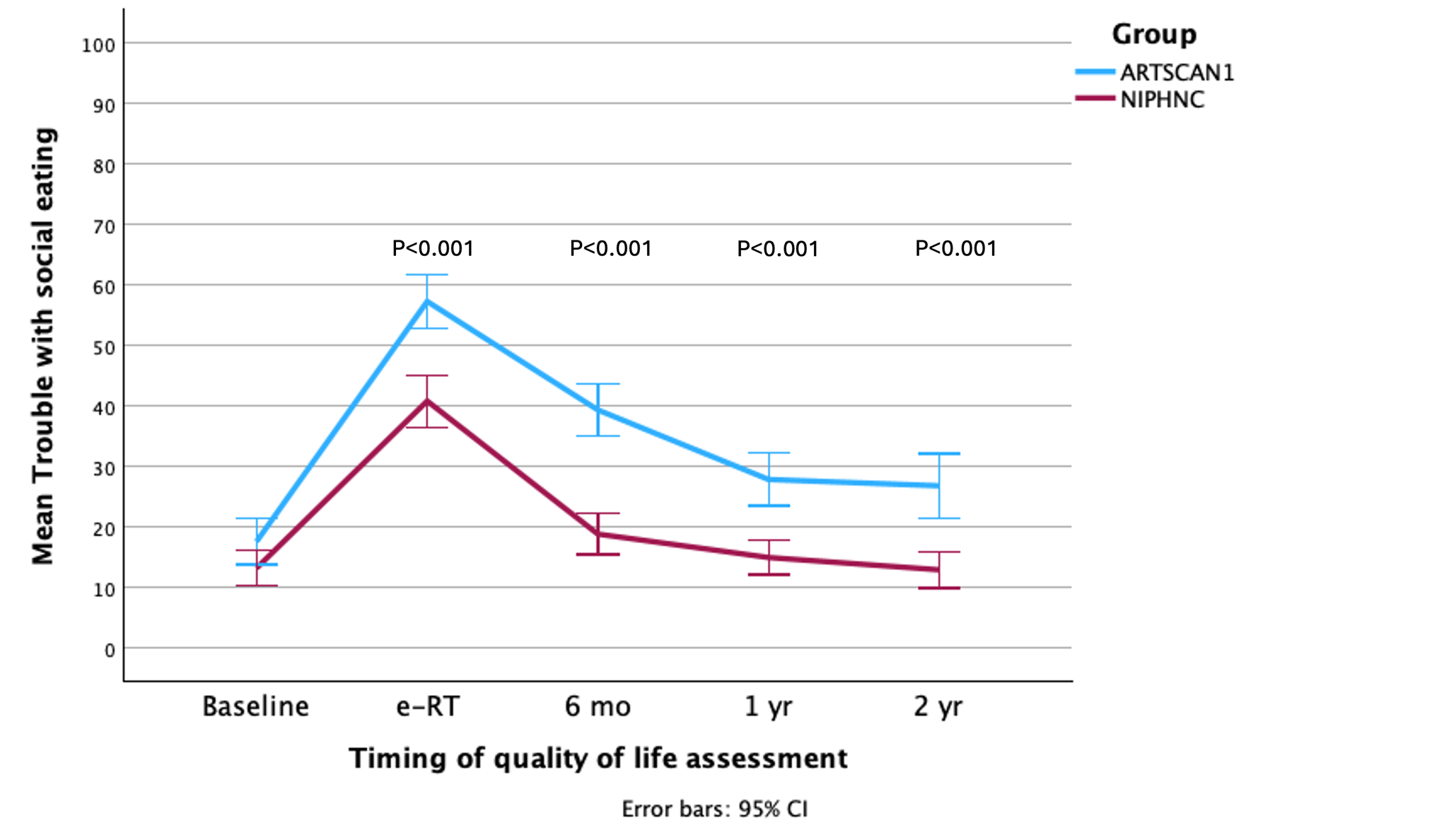  Figure 1K. Symptom scales of the EORTC QLQ- H&N35 (0-100 point scale) from baseline over 2 years reported by the ARTSCAN1 and NIPHNC groups. Mean values based on patients answering the questionnaire. Higher scores indicate more severe symptoms or impairments. Only significant P-values are reported. |
| 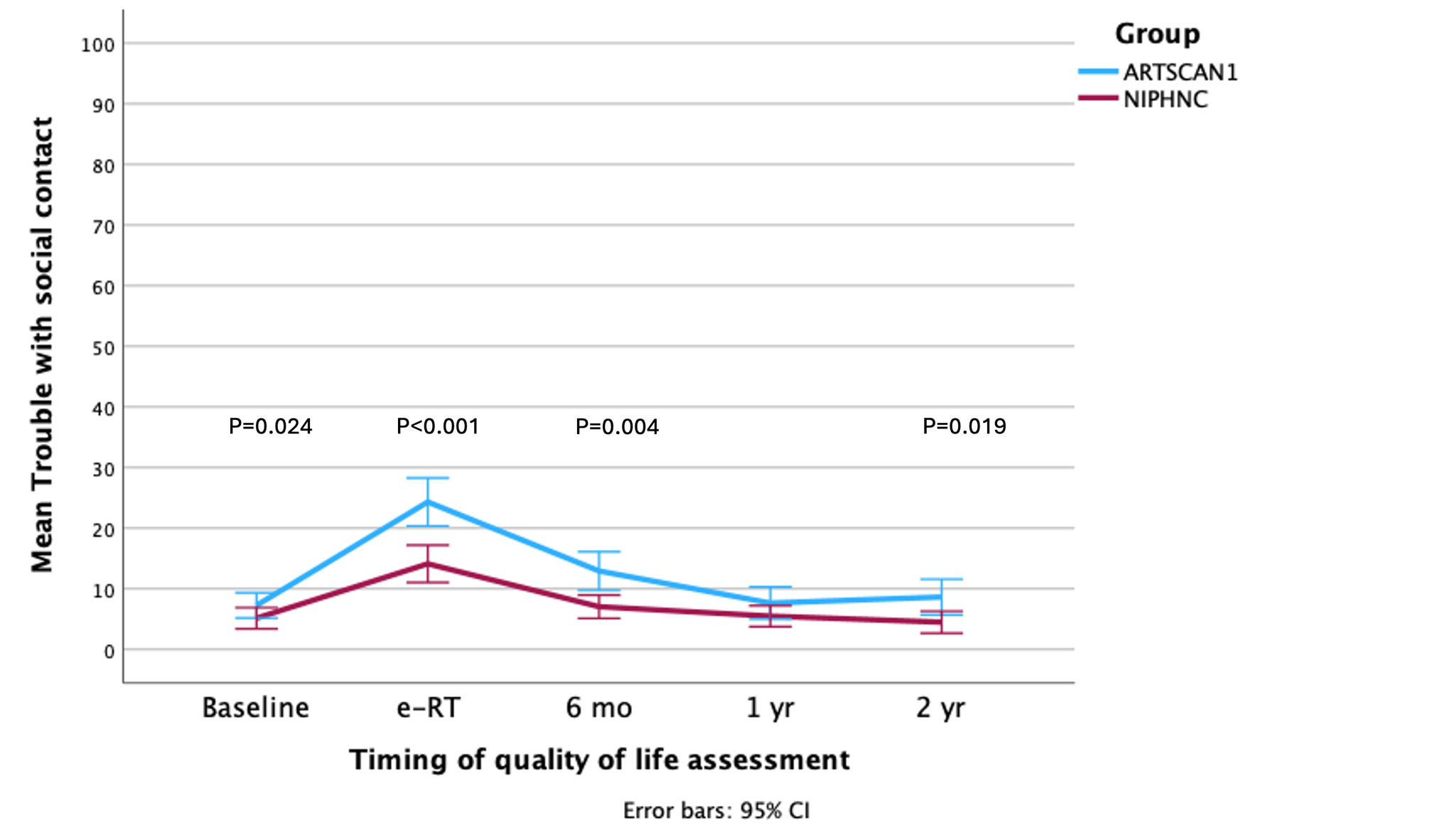  Figure 1L. Symptom scales of the EORTC QLQ- H&N35 (0-100 point scale) from baseline over 2 years reported by the ARTSCAN1 and NIPHNC groups. Mean values based on patients answering the questionnaire. Higher scores indicate more severe symptoms or impairments. Only significant P-values are reported. |
| 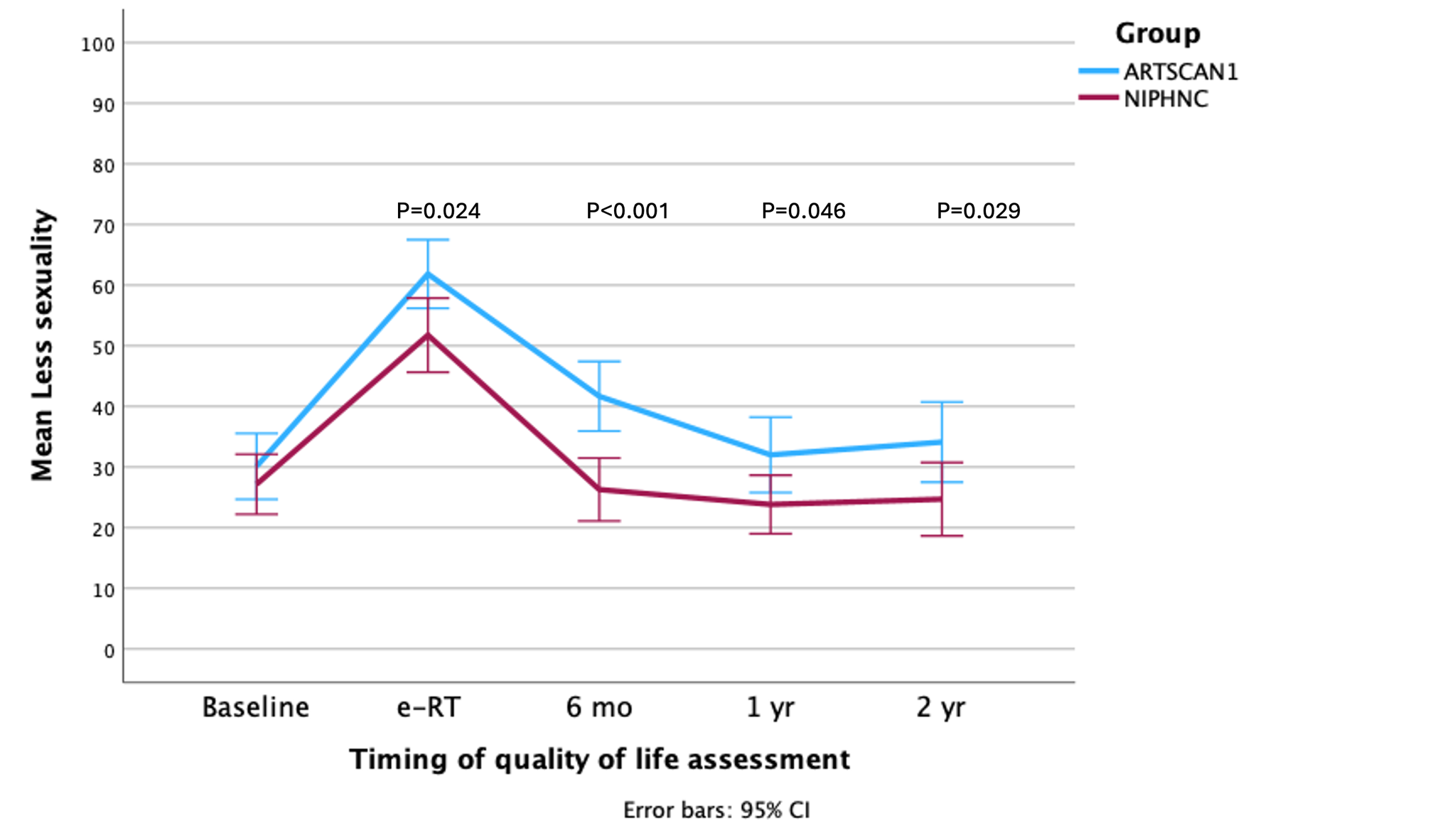  Figure 1M. Symptom scales of the EORTC QLQ- H&N35 (0-100 point scale) from baseline over 2 years reported by the ARTSCAN1 and NIPHNC groups. Mean values based on patients answering the questionnaire. Higher scores indicate more severe symptoms or impairments. Only significant P-values are reported. |
| 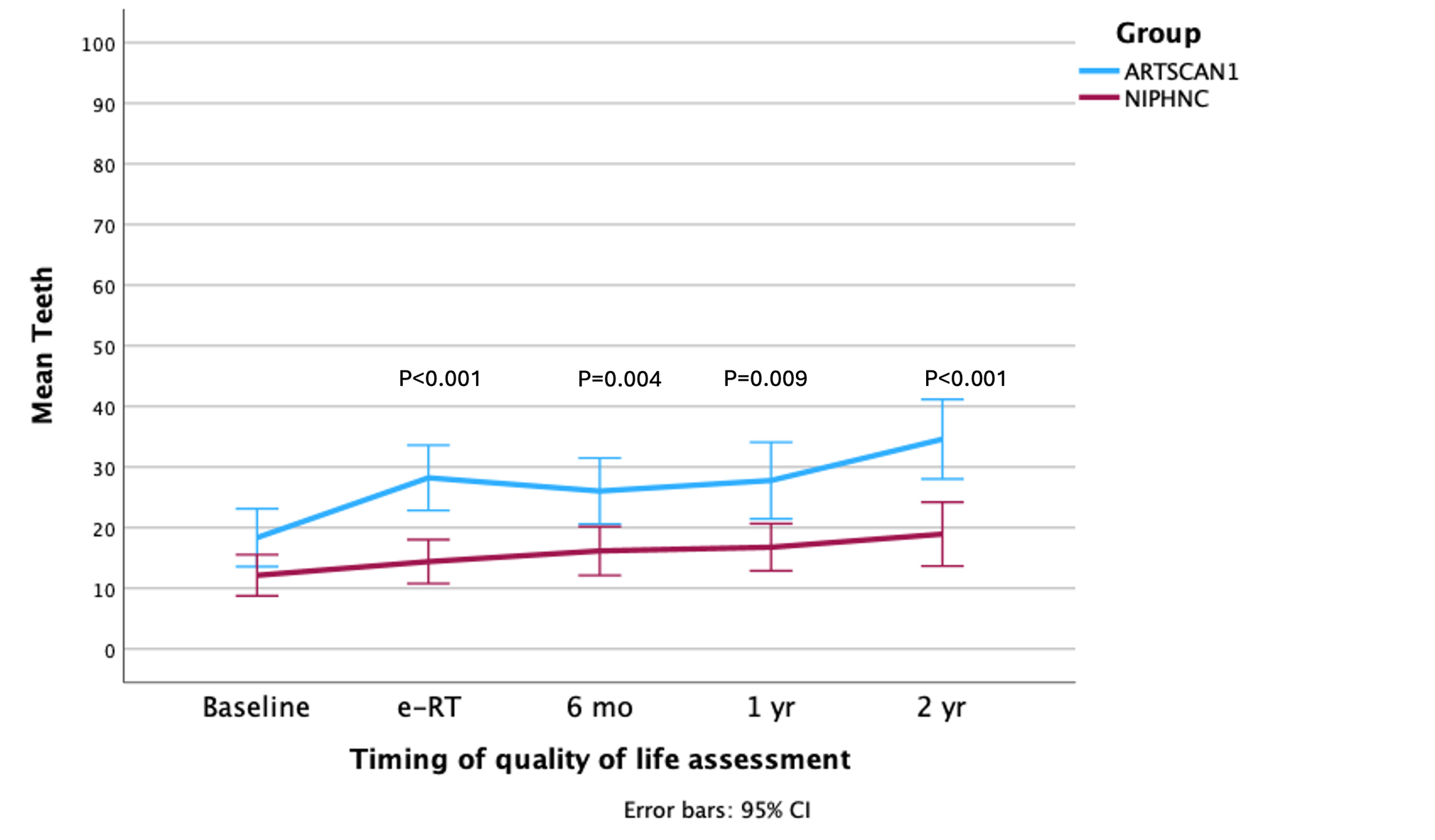  Figure 1N. Symptom scales of the EORTC QLQ- H&N35 (0-100 point scale) from baseline over 2 years reported by the ARTSCAN1 and NIPHNC groups. Mean values based on patients answering the questionnaire. Higher scores indicate more severe symptoms or impairments. Only significant P-values are reported. |
| 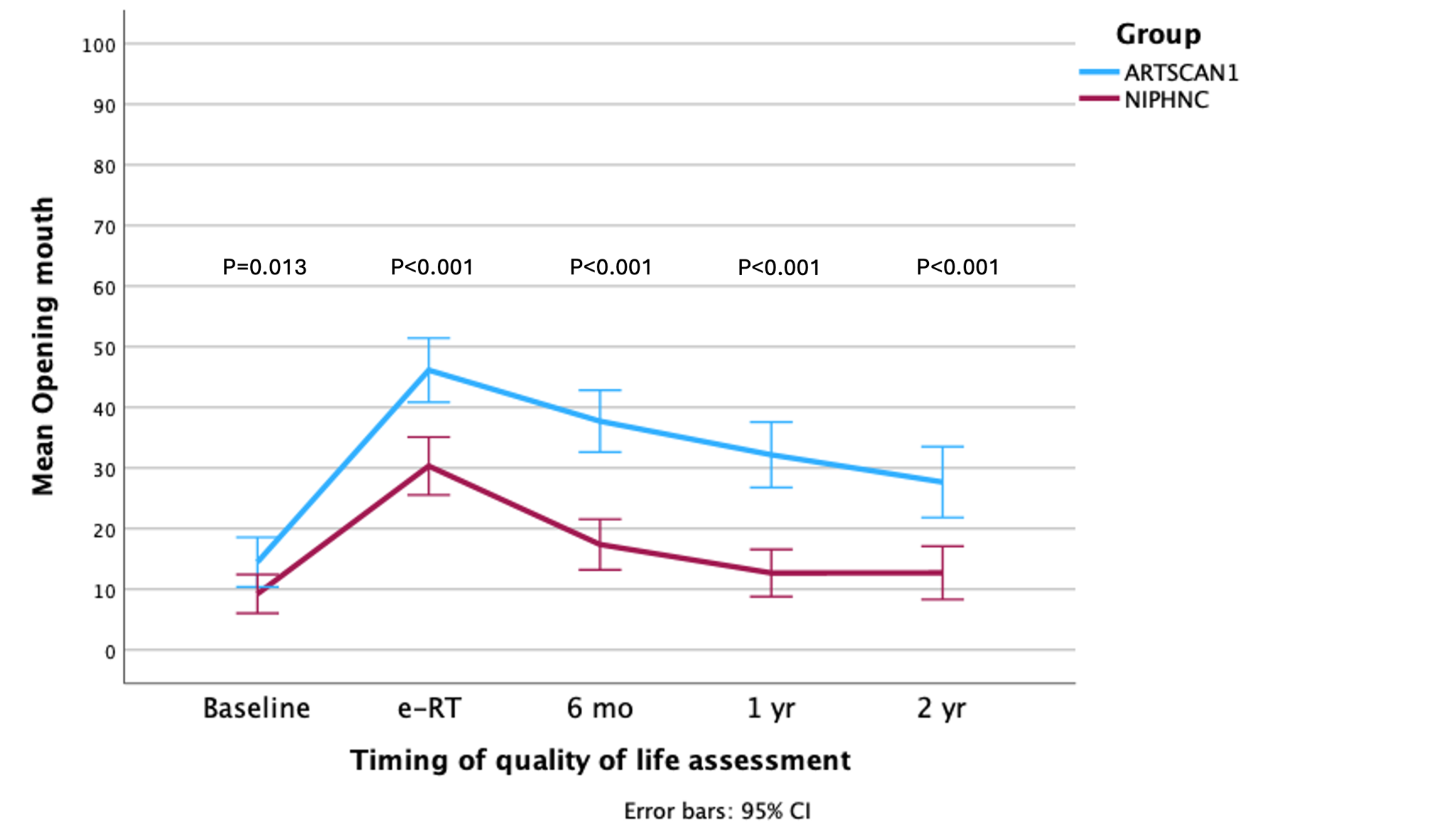  Figure 1O. Symptom scales of the EORTC QLQ- H&N35 (0-100 point scale) from baseline over 2 years reported by the ARTSCAN1 and NIPHNC groups. Mean values based on patients answering the questionnaire. Higher scores indicate more severe symptoms or impairments. Only significant P-values are reported. |
| 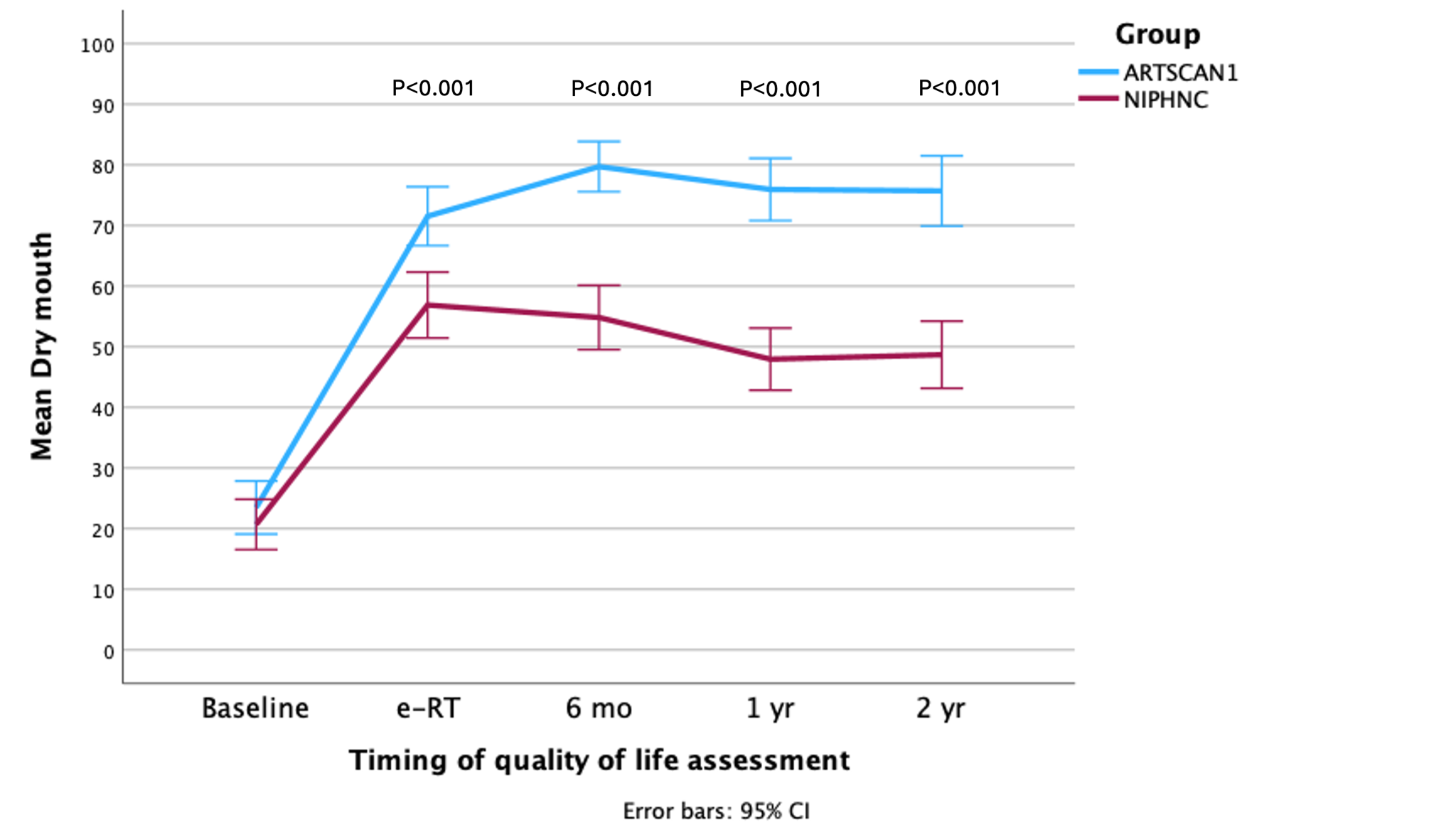  Figure 1P. Symptom scales of the EORTC QLQ- H&N35 (0-100 point scale) from baseline over 2 years reported by the ARTSCAN1 and NIPHNC groups. Mean values based on patients answering the questionnaire. Higher scores indicate more severe symptoms or impairments. Only significant P-values are reported. |
| 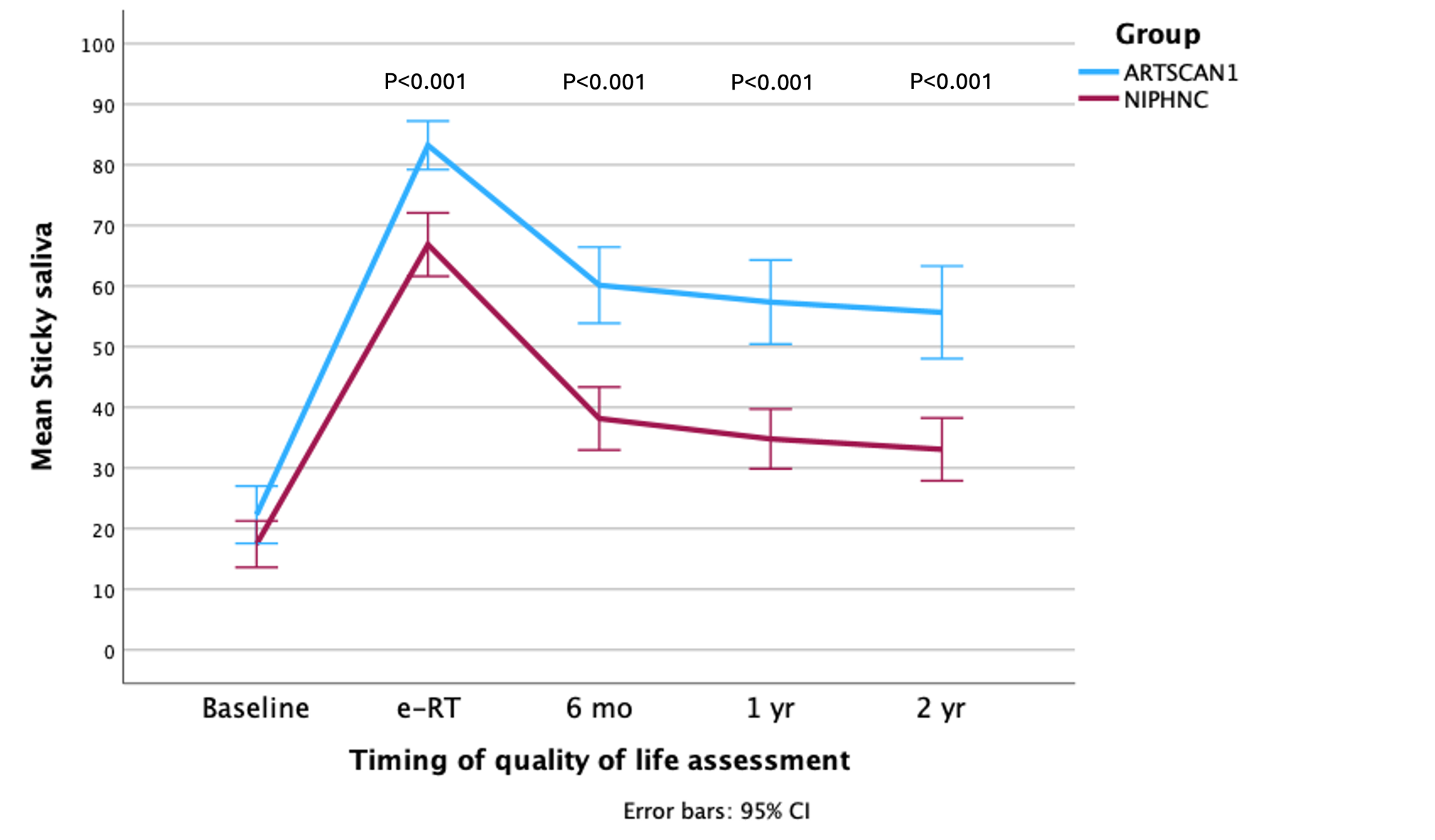  Figure 1Q. Symptom scales of the EORTC QLQ- H&N35 (0-100 point scale) from baseline over 2 years reported by the ARTSCAN1 and NIPHNC groups. Mean values based on patients answering the questionnaire. Higher scores indicate more severe symptoms or impairments. Only significant P-values are reported. |
| 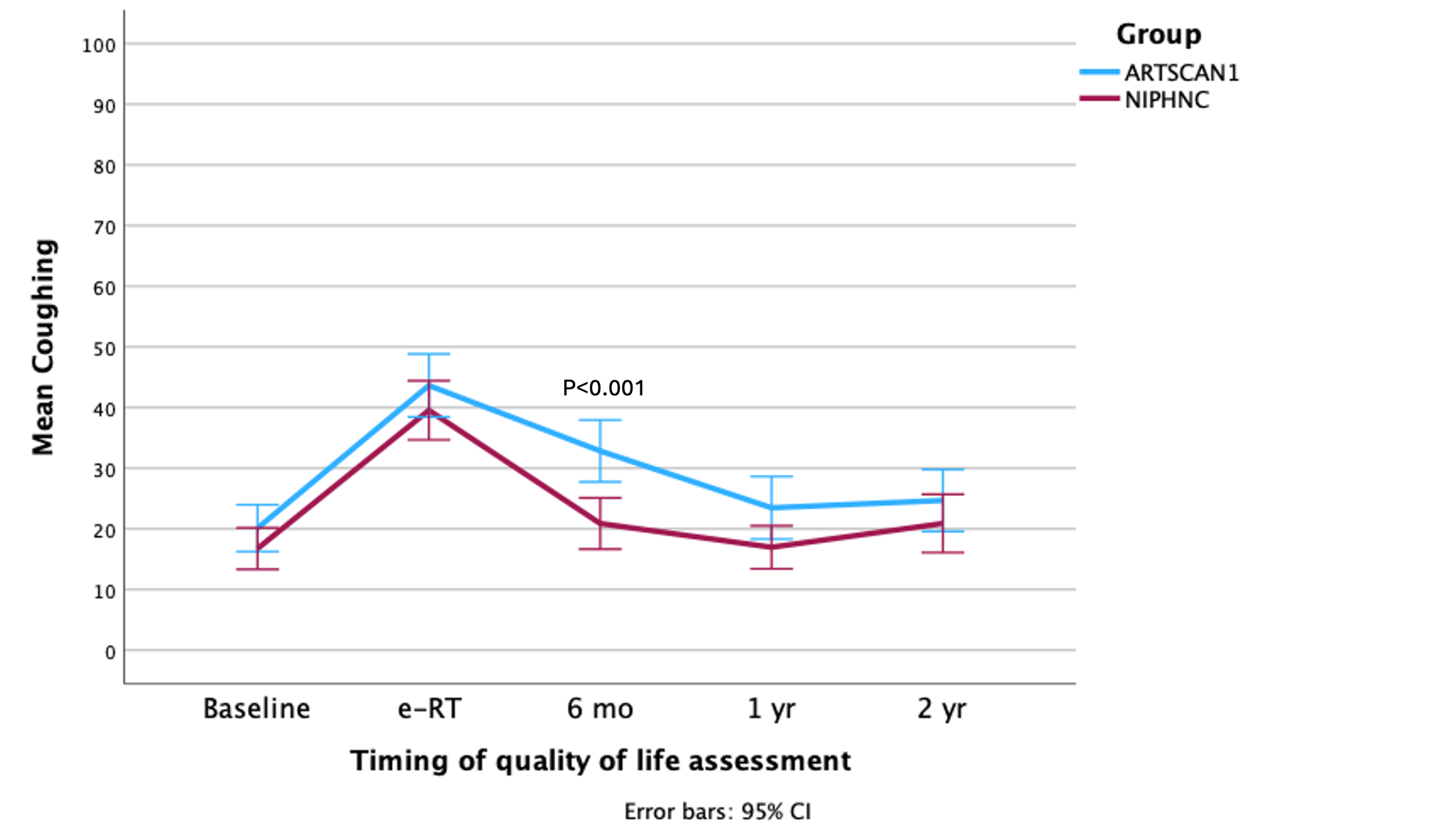  Figure 1R. Symptom scales of the EORTC QLQ- H&N35 (0-100 point scale) from baseline over 2 years reported by the ARTSCAN1 and NIPHNC groups. Mean values based on patients answering the questionnaire. Higher scores indicate more severe symptoms or impairments. Only significant P-values are reported. |
| 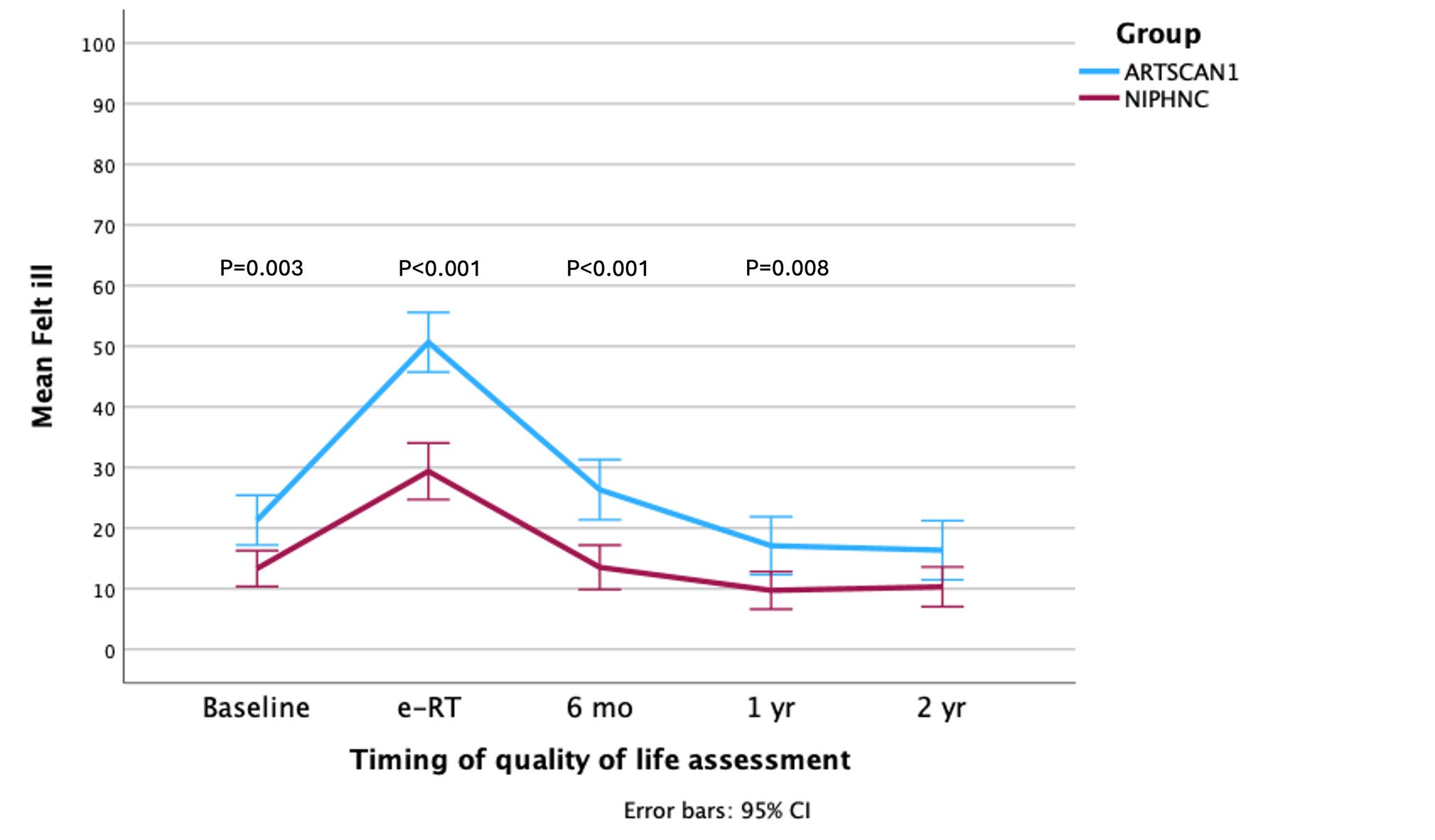  Figure 1S. Symptom scales of the EORTC QLQ- H&N35 (0-100 point scale) from baseline over 2 years reported by the ARTSCAN1 and NIPHNC groups. Mean values based on patients answering the questionnaire. Higher scores indicate more severe symptoms or impairments. Only significant P-values are reported. |
| 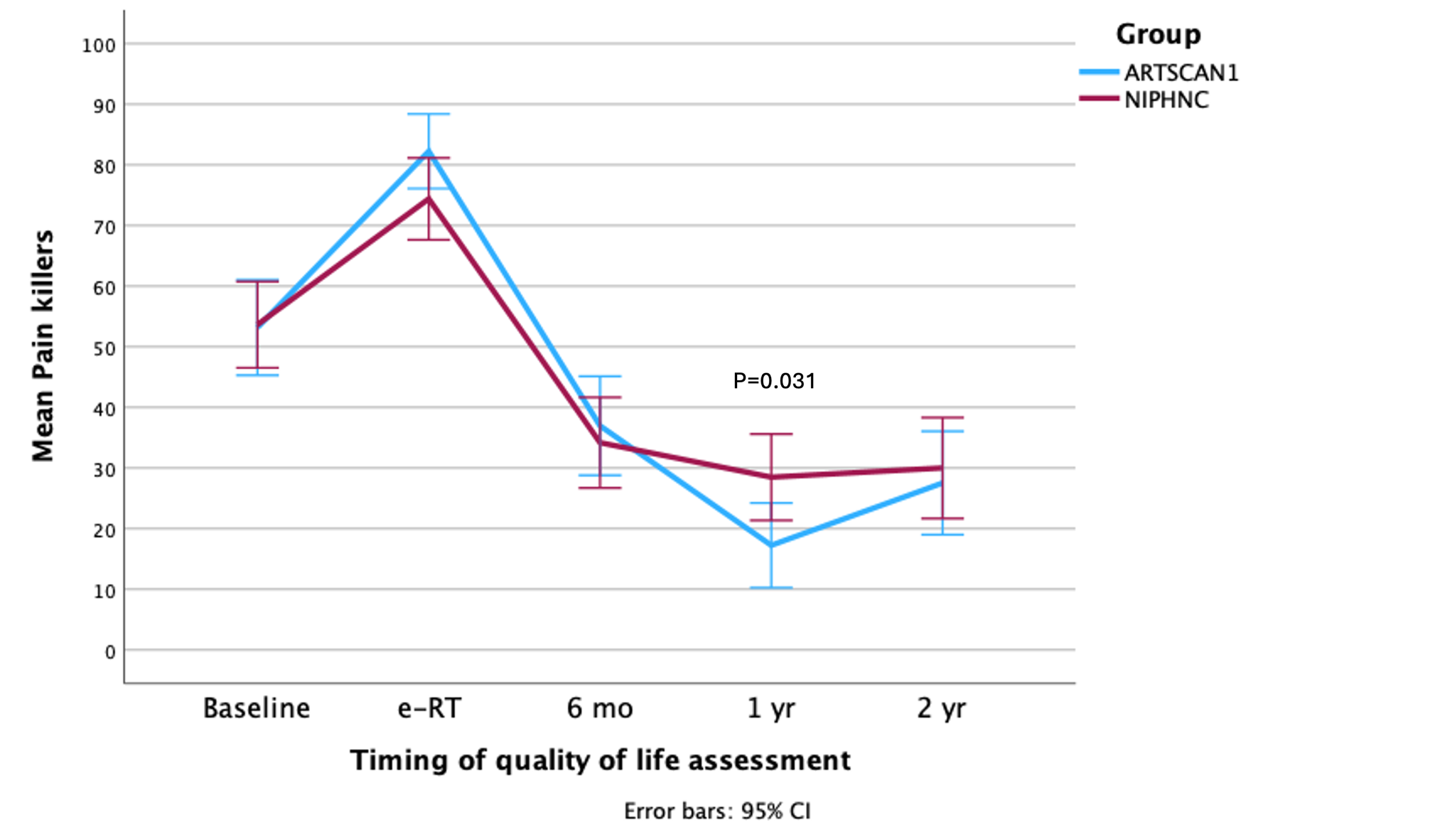  Figure 1T. Symptom scales of the EORTC QLQ- H&N35 (0-100 point scale) from baseline over 2 years reported by the ARTSCAN1 and NIPHNC groups. Mean values based on patients answering the questionnaire. Higher scores indicate more severe symptoms or impairments. Only significant P-values are reported. |
| 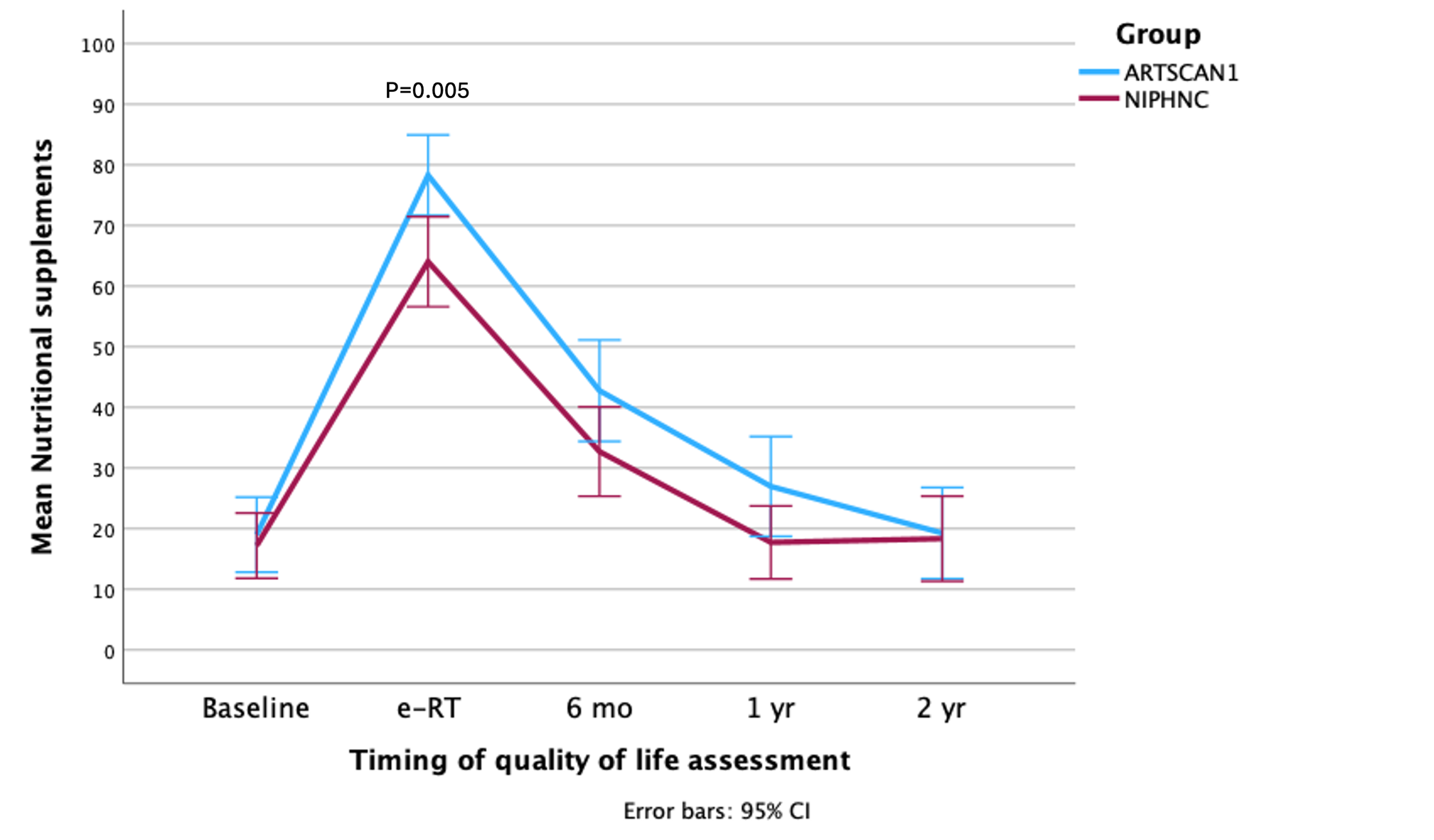  Figure 1U. Symptom scales of the EORTC QLQ- H&N35 (0-100 point scale) from baseline over 2 years reported by the ARTSCAN1 and NIPHNC groups. Mean values based on patients answering the questionnaire. Higher scores indicate more severe symptoms or impairments. Only significant P-values are reported. |
| 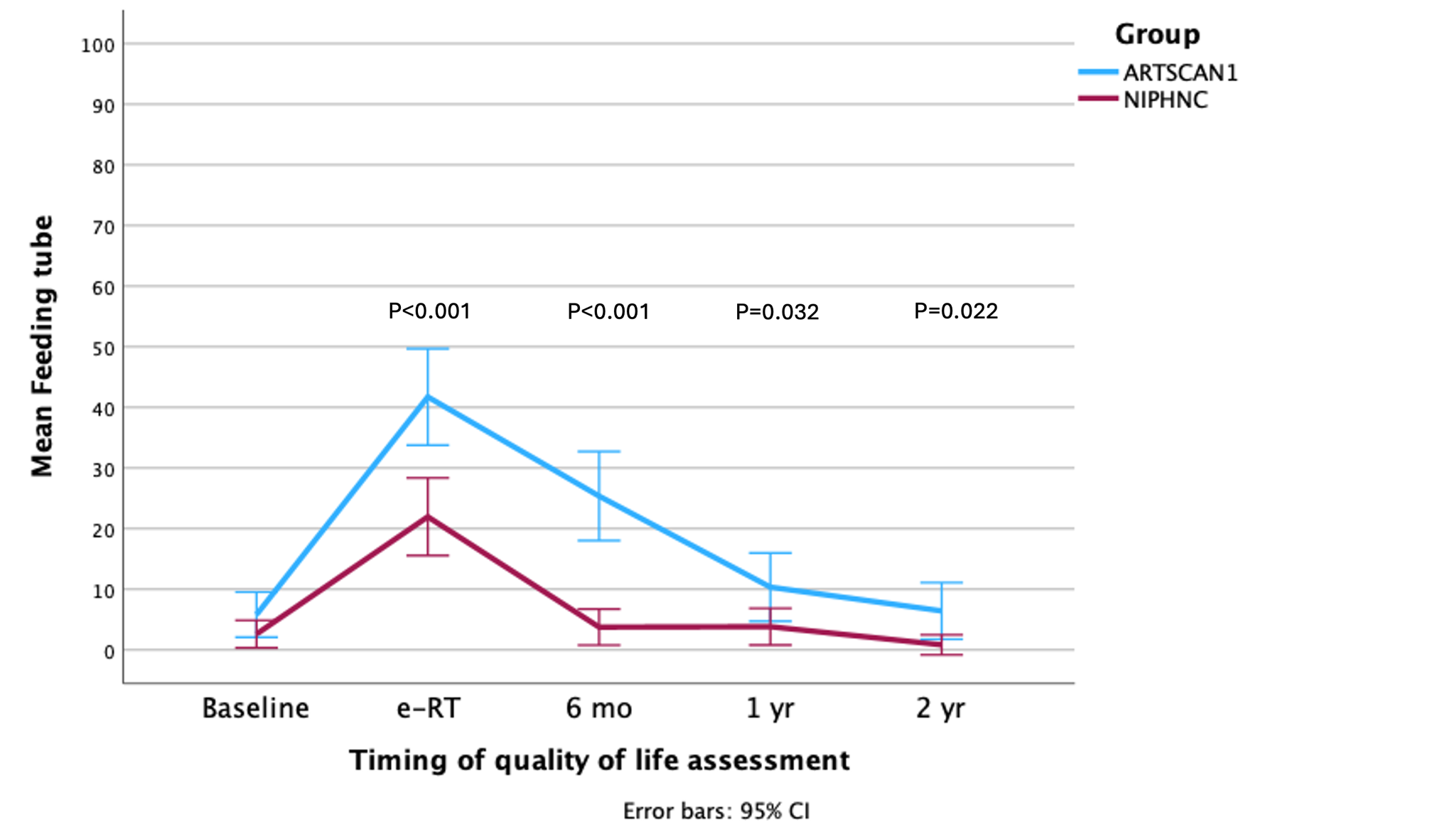  Figure 1V. Symptom scales of the EORTC QLQ- H&N35 (0-100 point scale) from baseline over 2 years reported by the ARTSCAN1 and NIPHNC groups. Mean values based on patients answering the questionnaire. Higher scores indicate more severe symptoms or impairments. Only significant P-values are reported. |
| 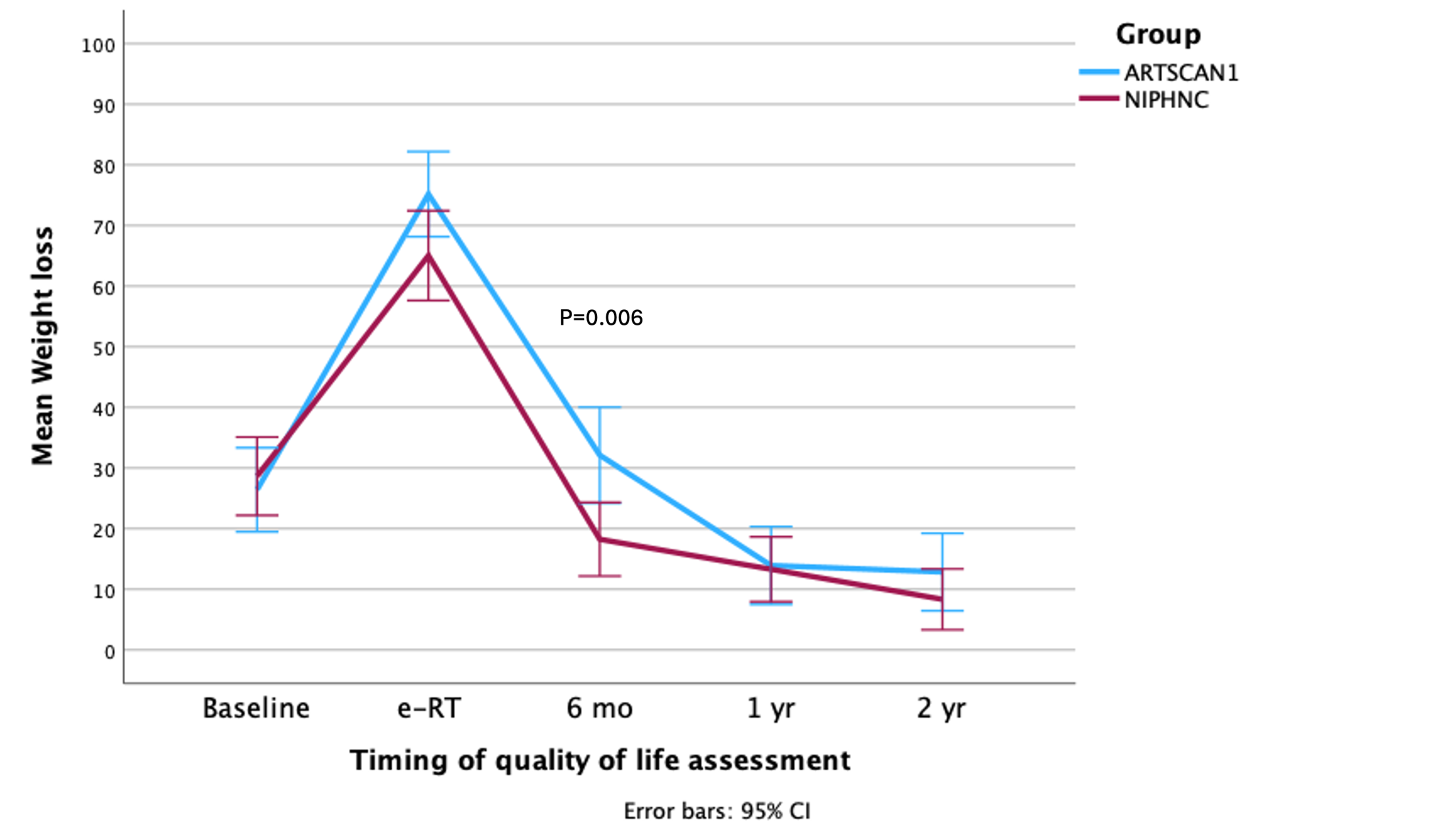  Figure 1Z. Symptom scales of the EORTC QLQ- H&N35 (0-100 point scale) from baseline over 2 years reported by the ARTSCAN1 and NIPHNC groups. Mean values based on patients answering the questionnaire. Higher scores indicate more severe symptoms or impairments. Only significant P-values are reported. |
| 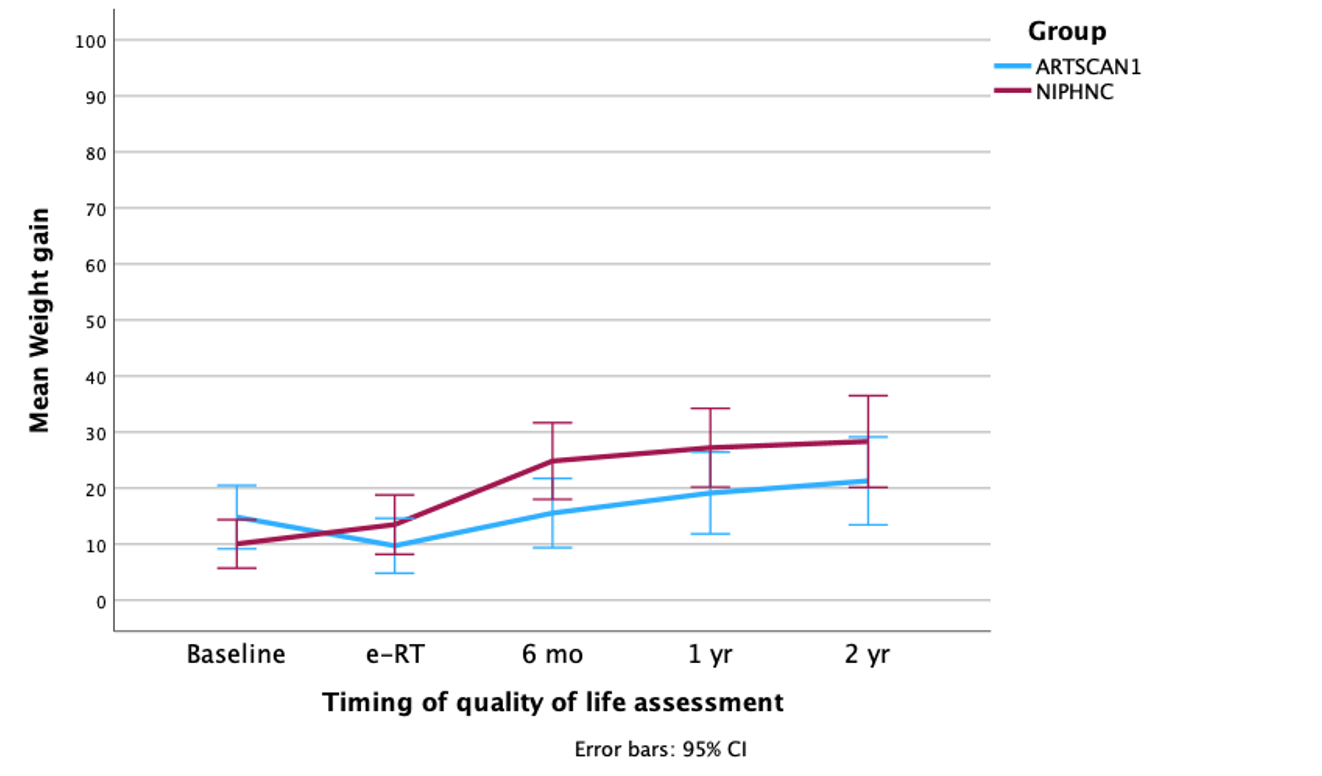  Figure 1X. Symptom scales of the EORTC QLQ- H&N35 (0-100 point scale) from baseline over 2 years reported by the ARTSCAN1 and NIPHNC groups. Mean values based on patients answering the questionnaire. Higher scores indicate more severe symptoms or impairments. Only significant P-values are reported. |
