# Supplementary material for: Patient-reported outcomes in oropharyngeal cancer: comparing two cohorts with different treatment protocols
Source: Support Care Cancer. 2026 May 13;34(6):536. doi: 10.1007/s00520-026-10763-2 (PMC13171772; doi:10.1007/s00520-026-10763-2)
Supplement: Supplementary file 2 — Supplementary file2 (DOCX 56 kb) [file 520_2026_10763_MOESM2_ESM.docx]

Supplementary material, table 1, univariate and multivariable linear regression at the end of RT and 2-year follow-up on the (EORTC-QLQ-H&N35) module and the Hospital Anxiety and Depression Scale (HADS)

|  |  | FOLLOW UP | | | |
| --- | --- | --- | --- | --- | --- |
|  |  | END-OF RT | | 2-YEAR | |
| Dependent | **Independent** | p-value  (univariate)^a^ | p-value  (model)^b^ | p-value  (univariate)^a^ | p-value  (model)^b^ |
| Pain | Smoker | 0.686 |  | 0.534 |  |
|  | RT-technique | 0.774 |  | 0.285 |  |
|  | Sex | 0.024 |  | 0.055 |  |
|  | Age | 0.005 | **0.012** | 0.865 |  |
|  | Neck dissection | 0.898 |  | 0.465 |  |
|  | SACT | 0.314 |  | 0.077 |  |
|  | Stage | 0.547 |  | 0.915 |  |
| Swallowing | Smoker | 0.336 |  | 0.075 |  |
|  | RT-technique | 0.655 |  | 0.237 |  |
|  | Sex | 0.175 |  | 0.961 |  |
|  | Age | 0.015 |  | 0.841 |  |
|  | Neck dissection | 0.967 |  | 0.476 |  |
|  | SACT | 0.346 |  | 0.169 |  |
|  | Stage | 0.134 |  | 0.365 |  |
| Senses problems | Smoker | 0.116 |  | 0.571 |  |
|  | RT-technique | 0.668 |  | 0.263 |  |
|  | Sex | 0.838 |  | 0.681 |  |
|  | Age | 0.140 |  | 0.253 |  |
|  | Neck dissection | 0.860 |  | 0.327 |  |
|  | SACT | 0.042 |  | 0.023 |  |
|  | Stage | 0.944 |  | 0.970 |  |
| Speech problems | Smoker | 0.591 |  | 0.057 |  |
|  | RT-technique | 0.312 |  | 0.637 |  |
|  | Sex | 0.673 |  | 0.984 |  |
|  | Age | <00.001 | **0.001** | 0.737 |  |
|  | Neck dissection | 0.199 |  | 0.550 |  |
|  | SACT | 0.521 |  | 0.301 |  |
|  | Stage | 0.010 | **0.021** | 0.044 | **0.047** |
| Trouble with social eating | Smoker | 0.400 |  | 0.014 | **0.024** |
|  | RT-technique | 0.817 |  | 0.843 |  |
|  | Sex | 0.533 |  | 0.822 |  |
|  | Age | 0.017 | **0.008** | 0.250 |  |
|  | Neck dissection | 0.744 |  | 0.504 |  |
|  | SACT | 0.027 | **0.012** | 0.004 | **0.008** |
|  | Stage | 0.666 |  | 0.360 |  |
| Trouble with social contact | Smoker | 0.681 |  | 0.041 |  |
|  | RT-technique | 0.265 |  | 0.512 |  |
|  | Sex | 0.016 | **0.031** | 0.713 |  |
|  | Age | 0.023 | **0.046** | 0.855 |  |
|  | Neck dissection | 0.212 |  | 0.695 |  |
|  | SACT | 0.390 |  | 0.322 |  |
|  | Stage | 0.846 |  | 0.127 |  |
| Less sexuality | Smoker | 0.269 |  | 0.612 |  |
|  | RT-technique | 0.040 |  | 0.276 |  |
|  | Sex | 0.162 |  | 0.392 |  |
|  | Age | 0.025 |  | 0.296 |  |
|  | Neck dissection | 0.277 |  | 0.427 |  |
|  | SACT | 0.709 |  | 0.229 |  |
|  | Stage | 0.357 |  | 0.231 |  |
| Teeth | Smoker | 0.008 | **0.014** | 0.012 |  |
|  | RT-technique | 0.567 |  | 0.273 |  |
|  | Sex | 0.022 |  | 0.151 |  |
|  | Age | 0.420 |  | 0.820 |  |
|  | Neck dissection | 0.214 |  | 0.437 |  |
|  | SACT | 0.287 |  | 0.122 |  |
|  | Stage | 0.097 |  | 0.819 |  |
| Opening mouth | Smoker | 0.141 |  | 0.063 |  |
|  | RT-technique | 0.247 |  | 0.370 |  |
|  | Sex | 0.021 |  | 0.924 |  |
|  | Age | 0.002 | **0.005** | 0.237 |  |
|  | Neck dissection | 0.898 |  | 0.603 |  |
|  | SACT | 0.152 |  | 0.077 |  |
|  | Stage | 0.334 |  | 0.682 |  |
| Dry mouth | Smoker | 0.160 |  | 0.062 |  |
|  | RT-technique | 0.526 |  | 0.687 |  |
|  | Sex | 0.822 |  | 0.478 |  |
|  | Age | 0.198 |  | 0.754 |  |
|  | Neck dissection | 0.790 |  | 0.089 | **0.036** |
|  | SACT | 0.018 |  | 0.002 | **0.003** |
|  | Stage | 0.588 |  | 0.997 |  |
| Sticky saliva | Smoker | 0.134 |  | 0.031 |  |
|  | RT-technique | 0.088 |  | 0.044 |  |
|  | Sex | 0.799 |  | 0.778 |  |
|  | Age | 0.171 |  | 0.623 |  |
|  | Neck dissection | 0.122 |  | 0.081 |  |
|  | SACT | 0.204 |  | 0.074 |  |
|  | Stage | 0.464 |  | 0.796 |  |
| Coughing | Smoker | 0.724 |  | 0.014 |  |
|  | RT-technique | 0.626 |  | 0.928 |  |
|  | Sex | 0.892 |  | 0.272 |  |
|  | Age | 0.097 |  | 0.245 |  |
|  | Neck dissection | 0.119 |  | 0.630 |  |
|  | SACT | 0.694 |  | 0.445 |  |
|  | Stage | 0.037 | **0.047** | 0.299 |  |
| Felt ill | Smoker | 0.369 |  | 0.081 |  |
|  | RT-technique | 0.104 |  | 0.332 |  |
|  | Sex | 0.142 |  | 0.024 |  |
|  | Age | <00.001 | **<0.001** | 0.883 |  |
|  | Neck dissection | 0.159 |  | 0.602 |  |
|  | SACT | 0.044 | **0.009** | 0.168 |  |
|  | Stage | 0.098 |  | 0.563 |  |
| Pain killers | Smoker | 0.647 |  | 0.311 |  |
|  | RT-technique | 0.469 |  | 0.249 |  |
|  | Sex | 0.283 |  | 0.036 |  |
|  | Age | 0.769 |  | 0.318 |  |
|  | Neck dissection | 0.354 |  | 0.540 |  |
|  | SACT | 0.840 |  | 0.158 |  |
|  | Stage | 0.574 |  | 0.190 |  |
| Nutritional supplements | Smoker | 0.088 |  | 0.112 |  |
|  | RT-technique | 0.453 |  | 0.411 |  |
|  | Sex | 0.169 |  | 0.266 |  |
|  | Age | 0.502 |  | 0.098 |  |
|  | Neck dissection | 0.958 |  | 0.662 |  |
|  | SACT | 0.477 |  | 0.791 |  |
|  | Stage | 0.217 |  | 0.620 |  |
| Feeding tube | Smoker | 0.858 |  | 0.130 |  |
|  | RT-technique | 0.912 |  | 0.869 |  |
|  | Sex | 0.841 |  | 0.485 |  |
|  | Age | 0.022 | **0.012** | 0.713 |  |
|  | Neck dissection | 0.254 |  | 0.944 |  |
|  | SACT | 0.913 |  | 0.440 |  |
|  | Stage | 0.032 | **0.018** | 0.350 |  |
| Weight loss | Smoker | 0.857 |  | <00.001 | **<0.001** |
|  | RT-technique | 0.667 |  | 0.588 |  |
|  | Sex | 0.362 |  | 0.778 |  |
|  | Age | 0.823 |  | 0.151 |  |
|  | Neck dissection | 0.243 |  | 0.686 |  |
|  | SACT | 0.164 |  | 0.058 |  |
|  | Stage | 0.020 |  | 0.217 |  |
| Weight gain | Smoker | 0.282 |  | 0.708 |  |
|  | RT-technique | 0.376 |  | 0.134 |  |
|  | Sex | 0.644 |  | 0.468 |  |
|  | Age | 0.472 |  | 0.815 |  |
|  | Neck dissection | 0.508 |  | 0.593 |  |
|  | SACT | 0.394 |  | 0.058 |  |
|  | Stage | 0.831 |  | 0.797 |  |
| Anxiety | Smoker | 0.041 |  | 0.283 |  |
|  | RT-technique | 0.720 |  | 0.673 |  |
|  | Sex | 0.065 |  | 0.043 |  |
|  | Age | 0.015 |  | 0.150 |  |
|  | Neck dissection | 0.927 |  | 0.207 |  |
|  | SACT | <00.001 | **0.018** | <00.001 | **<0.001** |
|  | Stage | 0.782 |  | 0.947 |  |
| Depression | Smoker | 0.143 |  | 0.786 |  |
|  | RT-technique | 0.690 |  | 0.807 |  |
|  | Sex | 0.283 |  | 0.268 |  |
|  | Age | 0.037 | **0.011** | 0.031 | **0.003** |
|  | Neck dissection | 0.592 |  | 0.356 |  |
|  | SACT | <00.001 | **<0.001** | <00.001 | **<0.001** |
|  | Stage | 0.783 |  | 0.261 |  |

RT-Radiotherapy

RT-technique- 3DCRT or IMRT/VMAT

SACT- Systemic anti-cancer treatment

Stage- Stage I-II or Stage III-IV

^a^Only independent variables with p-values <0.1 in the univariate analysis are included in the multivariate analysis.

^b^Only significant p-values <0.05 (in bold text) in the multivariable analysis model are reported.
